# The Influence of Social Isolation on Social Orientation, Sociability, Social Novelty Preference, and Hippocampal Parvalbumin-Expressing Interneurons in Peripubertal Rats – Understanding the Importance of Meeting Social Needs in Adolescence

**DOI:** 10.3389/fnbeh.2022.872628

**Published:** 2022-05-03

**Authors:** Milica Potrebić, Željko Pavković, Nela Puškaš, Vesna Pešić

**Affiliations:** ^1^Molecular Neurobiology and Behavior, Department of Neurobiology, Institute for Biological Research “Siniša Stanković”, National Institute of Republic of Serbia, University of Belgrade, Belgrade, Serbia; ^2^Institute of Histology and Embryology “Aleksandar Đ. Kostić”, Faculty of Medicine, University of Belgrade, Belgrade, Serbia

**Keywords:** adolescence, animal model, maturation, motivation, social needs, 3-chamber test, social odor

## Abstract

The fulfillment of belonging needs underlies a variety of behaviors. In order to understand how social needs unmet during maturation shape everyday life, we examined social motivation and cognition in peripubertal rats, as a rodent model of adolescence, subjected to social isolation (SI) during early and early-to-mid adolescence. The behavioral correlates of social orientation (social space preference), sociability (preference for social over non-social novelty), and social novelty preference (SNP) were examined in group-housed (GH) and single-housed (SH) rats in a 3-chamber test. The response to social odors was examined to gain insights into the developmental role of social odors in motivated social behavior. Differentiation between appetitive (number of visits/approaches) and consummatory (exploratory time) aspects of motivated social behavior was done to determine which facet of social motivation characterizes maturation when social needs are met and which aspect dominates when social needs are unsatisfied. The SI-sensitive parvalbumin-expressing interneurons (PVI) in the hippocampus were examined using immunohistochemistry. The main findings are the following: (1) in GH rats, the preference for social space is not evident regardless of animals’ age, while sociability becomes apparent in mid-adolescence strictly through consummatory behavior, along with complete SNP (appetitive, consummatory); (2) SH promotes staying in a social chamber/space regardless of animals’ age and produces an appetitive preference for it only in early-adolescent animals; (3) SH promotes sociability (appetitive, consummatory) regardless of the animals’ age and prevents the SNP; (4) the preference for a social odor is displayed in all the groups through consummatory behavior, while appetitive behavior is evident only in SH rats; (5) the response to social odors does not commensurate directly to the response to conspecifics; (6) SH does not influence PVI in the hippocampus, except in the case of early-adolescence when a transient decrease in the dentate gyrus is observed. These results accentuate the developmental complexity of social motivation and cognition, and the power of SI in adolescence to infringe social maturation at different functional levels, promoting appetitive behavior toward peers overall but harming the interest for social novelty. The findings emphasize the importance of the fulfillment of basic social needs in the navigation through the social world.

## Introduction

Individual behavior is modulated by global trends and extraordinary circumstances, such as the recent COVID-19 pandemic that involved measures of social distancing and the usage of digital technology that supports activities that would normally be performed in person (Marroquín et al., 2020). With the increasing use of digital media, particularly in adolescents, the decrease in direct social contacts threatens to become a widespread problem in the 21st century (Twenge et al., 2021). The fundamental maturational task of adolescence is the achievement of social competence, with peer acceptance and integration as the primary focus of social motivation (Crone and Dahl, 2012; Nelson et al., 2016). Stable and positive social contacts with peers are a prerequisite for the fulfillment of belonging needs (Baumeister and Leary, 1995), a core social motive that is thought to underlie a wide variety of social behaviors (Over, 2016). Thus, the question that arises is how subjects with unmet basic social needs interact with novel social contexts and navigate through the social world.

Previous research studying unmet social needs has focused on the effects of social isolation (SI) in animal models and on the neural correlates of loneliness and social exclusion in humans, bringing forward evidence that social interactions represent a primary reward and a basic need of social animals and that distinct neural mechanisms are implicated in the motivation to fulfill an unmet need versus the reward when that need is met (Tomova et al., 2021). On the other hand, an in-depth analysis of behavioral manifestations of unmet social needs concerning social motivation and cognition in adolescence has not yet been examined in a rodent model. Experiments on rats could yield important findings because they are generally more sociable than mice (Bryda, 2013; Ellenbroek and Youn, 2016) and therefore present, perhaps, a better model system for this basic biological question.

The use of animal models involves more extreme forms of social deprivation than those seen in humans, and findings undoubtedly suggest that adolescence, a highly conserved developmental stage common among mammalian species (Spear, 2004), is particularly affected by the deprivation of social needs (Orben et al., 2020) due to maturational advancements in cognitive, affective, and social capacities that occur at that time (Crone and Dahl, 2012). Rats form affective bonds through social interactions and create groups based on social experience (Ben-Ami Bartal et al., 2014). Our previous study showed that the outcome of SI during peripuberty in the rat model is dynamic, starting with the cluster of atypical early symptoms that then evolve into a syndrome that is delicate for the assessment (Potrebic et al., 2021), indicating that changes in social behavior in isolated may also be heterogeneous, depending on which the periods of adolescence are being burdened. Although it is widely accepted that SI in rodents generally increases motivation to seek out and engage with conspecifics (Tomova et al., 2021), whether and how it influences the response to a social novelty over social familiarity, an important dimension of social behavior on a daily basis, has not been examined in maturing animals.

Social motivation theory states three components of motivation, namely social orientation, social seeking and liking, and maintenance, while diminished social interest has been related to deficits in social cognition (Chevallier et al., 2012). The three-chamber (3CH) test has been widely used for the assessment of motivated social behavior in a rodent model, as it enables the evaluation of the preference for social over non-social novelty (sociability), as well as the preference for social novelty over social familiarity in experimental animals (Crawley, 2004). In this test, the stimulus animal is restricted, which limits overt forms of aggressive, sexual, and play-fighting behaviors that are possible in free-interaction paradigms. When considering the use of the 3CH test, we came across several methodological dilemmas that improve the research methodology and contribute to the correct interpretation of the findings.

First, the social orientation of the animal in the 3CH (in terms of the preference for social over non-social space and familiar over non-familiar space) has been widely equated with the activity directed toward a relative. The separation of these activities could help recognize the role of social orientation as a meaningful construct for understanding social adjustment (Jorgensen and Nelson, 2018) since the subject animal must first visit the social space, and then the intrinsic motivation to approach or avoid direct social interaction would dictate the outcome.

Second, since the preference for a novel conspecific is a natural tendency of mice and rats (Schweinfurth, 2020), the omission of such behavior in the second phase of the 3CH test has been widely used as a measure of compromised social recognition memory because a novel companion has to be recognized as such based on the memory of the familiar. Social memory is a hippocampus-dependent memory (Pena et al., 2014; Almeida-Santos et al., 2019), with the Cornu Ammonis 2 (CA2) subfield of the dorsal hippocampus playing an essential role (Hitti and Siegelbaum, 2014; Dominguez et al., 2019). Novelty preference, as a valid index of recognition memory, has been used in other memory tests based on the natural curiosity of the animal toward novelty, for instance, in the novel object recognition test (Antunes and Biala, 2012), but some authors have questioned this, highlighting the importance of the attachment to the familiar (Ennaceur, 2010). Importantly, there are findings that the absence of social novelty preference may represent a deliberate choice of a known social goal to meet the basic needs for belonging and familiarity, as was shown in cognitively able individuals with atypical social development (Chen et al., 2015). It should be noted that, in humans, belonging needs represent an important motivational mechanism underlying the own-group memory bias (Van Bavel et al., 2012).

Third, we found reasonable the consideration that the appetitive/wanting aspect of motivated behavior (assessed through the number of visits/approaches) should be separated from the consummatory/liking aspect of motivated behavior (assessed through the time spent in exploration/sniffing duration) since, as already highlighted (Templer et al., 2018), they capture different aspects of social motivation and are separable both psychologically and neurobiologically: dopamine and dopamine interactions with corticolimbic glutamate and other neurochemical systems activate ‘wanting,’ while opioid, endocannabinoid, and GABA-benzodiazepine neurotransmitter systems are important for generating pleasurable/liking reactions at specific sites in limbic structures called ‘hedonic hotspots’ (Berridge et al., 2009).

Fourth, making conclusions about between-group differences in preferences for a conspecific based solely on exploratory time and a number of approaches could bring forward confounding results as these parameters speak about the exploratory behavior of animals within one experimental group while both low and high explorers could have the same overall preference. Therefore, a calculation of the discrimination index or recognition/preference index (both measures render exactly the same statistical outcome; Akkerman et al., 2012) should be done, allowing the control of variability due to individual differences in exploratory activity and statistical determination of a group preference.

Next, we considered the role of social odors in the approach behavior of adolescent rats. Social odors in rodents, like vision and eye contact in humans (Kampe et al., 2001), represent stimuli with social relevance that are important for sensory processing of social information and approach behavior (Contestabile et al., 2021), while social recognition relies upon the integration of olfactory, auditory, and somatosensory cues, hence requiring active behavior of social stimuli (Haskal et al., 2020). However, when exactly a maturing rodent becomes able to integrate these stimuli has not been examined to date. Although it is known that aberrant sensory processing is a core aspect of atypical social behavior (Crane et al., 2009), little attention has been paid to the relationship between responses to social olfactory cues and a real peer in rodent models.

During adolescence, the brain of social mammals undergoes substantial changes in structure and function, for which reason it has been recognized as a sensitive period of brain development (Fuhrmann et al., 2015). It is characterized by heightened neural plasticity, which is during sensitive periods experience-expectant – an organism ‘expects’ to be exposed to a particular stimulus during this time (Fuhrmann et al., 2015). Recent findings have shown that parvalbumin-positive (PV^+^) interneurons (PVIs), a major type of inhibitory neurons in the brain, require juvenile social experience to establish adult social behavior, particularly those in the prefrontal cortex (Bicks et al., 2020). Although the PVIs are crucial for the mnemonic functions of the hippocampus (Lee et al., 2016; Zaletel et al., 2016; Ognjanovski et al., 2017; Dominguez et al., 2019), a brain region also important for social mapping in space (Schafer and Schiller, 2018), the influence of social experience (isolation) on the hippocampal PVIs has not been examined in maturing rats. It should be noted that the term “social space” does not merely encompass social stimuli in physical space but also constructs of social affiliations that are, as cognitive maps, represented by the hippocampus and which, according to literature mainly, involve CA1 and CA2 subfields (Leblanc and Ramirez, 2020). The hippocampal PVIs have been recognized as a population of neurons especially vulnerable to chronic stress, which may be of key importance in the development of mood disorders (Zaletel et al., 2016). However, the findings in adult animals indicate that 3 weeks of SI produce a significant decrease in PV immunoreactivity (i.e., in the number of PV^+^ neurons) in all hippocampal subfields, along with a low level of serum corticosterone, suggesting that psychosocial isolation is *per se* sufficient to cause a decrease in PV immunoreactivity (Filipovic et al., 2013, 2018). Moreover, reduced PV expression (the number of PV^+^ neurons) has been reported in human brain samples and mouse models of autism spectrum disorders (the main characteristic of which is diminished social motivation, Chevallier et al., 2012), which influences network properties by shifting excitation/inhibition balance toward enhanced inhibition (Filice et al., 2016, 2020). Bearing in mind the role of the hippocampus in social mapping and learning in space (Leblanc and Ramirez, 2020) and the sensitivity of hippocampal PVIs to SI in the adult rat brain (Filipovic et al., 2013, 2018), we considered conducting an immunohistochemical analysis of the PVIs in the hippocampal subfields to assess the relation of the findings with the spatial behavior of the animals in the 3CH test, as well as to consolidate age-related outcomes of this adverse social experience. As indicated in a recent review (Leblanc and Ramirez, 2020), social modulation of learning and memory is an emerging field of neuroscience, and both procedures and measured variables are important for understanding principles and mechanisms.

Considering all of the above mentioned, the present study aimed to examine the influence of early-adolescent and early-to-mid-adolescent SI on motivated social behavior and the hippocampal PVI in peripubertal male rats as the rodent model of adolescence. Male rats were used in view of the findings that the influence of peer-related stimuli on the reward system and adolescent decision making (Albert et al., 2013) is modulated by gender, i.e., exists only in males (Defoe et al., 2020). The behavioral correlates of social orientation, sociability, and social novelty preference were addressed using alive animals (the three-chamber test, Templer et al., 2018) as well as social odors as cues to gain insights into the developmental role of social odors in motivated social behavior (Haskal et al., 2020; Contestabile et al., 2021). Appetitive (wanting) and consummatory (liking) behaviors were assessed simultaneously because they capture different aspects of social interaction (Templer et al., 2018), and nothing is known about their maturational trajectories and susceptibility to a social context. Both parameters of exploratory activity and preference indexes were analyzed in detail in order to gain insights into maturational changes in exploratory activity as a basis for calculating discrimination indexes. The PVIs were analyzed in all hippocampal subfields, using staining for PV and a Purkinje cell protein 4 (PCP4) to delineate CA2/CA3 and CA2/CA1 borders. Given that adolescence is preserved among social mammalian species and that every aspect of adolescent behavior has a biological basis, such a detailed analysis made it possible to discern cross-species similarities in social motivation and cognition, thus reducing the gap between rodent and human research and improving knowledge of evolutionary conserved neurobehavioral consequences of unmet social needs.

## Materials and Methods

### Animals

Experiments were conducted on 29-days-old male Wistar Han rats, which were kept under standard housing conditions: a room temperature 22 ± 1°C, relative humidity 50 ± 5%, 12 h light/dark cycle with lights on at 7:00 am, standard cages of 425 mm (L) × 265 mm (W) × 180 (H) mm with enclosure size 800 cm × 800 cm (European standard Type 3H, ZOONLAB) made from transparent plastic, autoclaved wood shavings as bedding material provided in sufficient quantity to cover the floor to a depth of 2 cm, and standard chow and tap water provided *ad libitum*.

The rats used as stimuli (14 in total) in the 3CH test (demonstrator rats) were additional animals of the same age and sex that were used only for the purpose of this test. They were kept in standard group housing conditions (3–4 per cage).

All animal procedures complied with the policies on animal welfare with EU Directive 2010/63/EU and were approved by the Ethical Committee of the Institute (01-01/19) and by the National Ethics Research Committee (323-07-05339/2020-05).

### Experimental Procedure

The litter was a biological replicate, with a total of 23 litters used in the experiment. At P29, male rats from the same litter (weaned and divided by sex at P21) were subjected to separation in the way that three rats were randomly chosen for further group housing (GH) and one for single housing (SH; Potrebic et al., 2021). In the GH rats, one animal per cage was randomly selected to further represent the group in all individual behavioral tests and immunohistochemical analysis (labeling was done using a non-toxic permanent marker on the tail). The cages were placed side by side so rats could smell, see, and hear each other but interact socially only in the group cages. No cages containing female rats were placed near the cages with experimental male rats (rather, they were put on the adjacent shelves in the same room, at a distance of 2 m).

**FIGURE 1:**
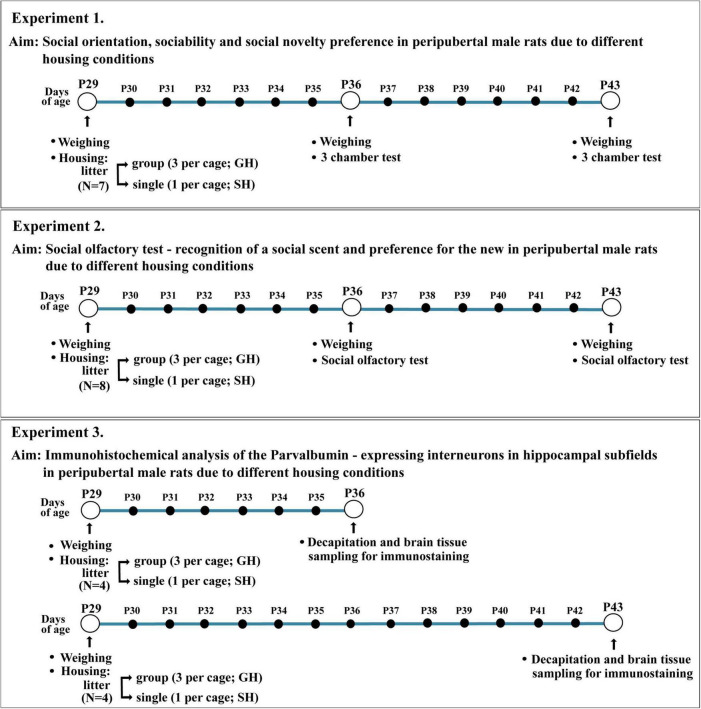
Schematic presentation of the experimental design.

The schematic presentation of the experimental design is given in Figure 1. Three experiments were performed, each with independent cohorts/litters of experimental animals: 7 for experiment 1, 8 for experiment 2, and 8 (i.e., 2 × 4) for experiment 3. Thus, the number of animals per group was *n* = 7 in experiment 1 (3CH test), *n* = 8 per group in experiment 2 (social olfactory test), and *n* = 4 per group in experiment 3 (immunohistochemical analysis of the Parvalbumin – expressing interneurons in hippocampal subfields). The behavioral testing (experiments 1 and 2) and immunohistochemical analyses were performed after the first and second weeks of defined housing, i.e., in rats approximately at the end of early-adolescent period/the onset of the mid-adolescent period and in the mid-adolescent period of development, respectively [puberty in male Wistar rats typically occurs on P41 ± 1 day; rodent adolescence is separated into early-adolescence (P21–P34), mid-adolescence (P34–P46), and late-adolescence (P46–P59); an ontogenic period that in rodents covers 7–10 days before puberty and a few days thereafter is also termed peri-adolescence; Burke and Miczek, 2014].

#### Experiment 1: Social Orientation, Sociability, and Social Novelty Preference

The 3-chamber test (3CHT) was used to assess social tendencies in rats that mature under different housing conditions, considering social motivation theory (Chevallier et al., 2012) and already existing findings on the wanting and liking components of social motivation in rats (Templer et al., 2018).

##### Apparatus

The 3CH box was made (Elunit, Belgrade, Serbia) considering the dimensions of the UgoBasile Sociability Cage for rats (120 cm × 40 cm × 40 cm). Chambers (40 cm × 40 cm each) were separated by two plastic doors, opening into the central compartment. The left and right compartments contained the cylindrical cage construction (25 cm × 15 cm; the grid bars had a diameter of 3 mm and were 7 mm apart), allowing close interaction between the testing subjects.

##### Test Procedure

Before behavioral testing, all animals were habituated to the testing room for 1 h, and all testing took place between 9:00 am and 2:00 pm. The testing was performed as described by Templer et al. (2018), with small modifications regarding the habituation procedure. During *the habituation phase*, the subject rat was placed in the central compartment of the 3CH and was allowed to freely explore the empty apparatus for 10 min. Doors to the left and right chambers remained open as a means to completely familiarize the animal with the novel environment (Zanos et al., 2014). No other rats or empty cages were present during the habituation phase. After this session, the subject rat was briefly returned to the home cage, and two cylindrical cage constructions were placed in the left and right compartments of the 3CH, one holding an unfamiliar rat (novel conspecific 1, N1), while the other was unoccupied (empty cage, O). The subject animal was then gently placed in the middle chamber to freely explore for an additional 10 min, and this session represented the *sociability phase*. Thereafter, the subject rat was briefly returned to the home cage and the second unfamiliar rat (novel conspecific 2, N2) was placed in the empty cylindrical cage, while the N1 remained in its cylindrical cage (representing socially familiar conspecific). Then, the subject rat was gently placed in the middle chamber and allowed to freely explore the 3CH for the next 10 min, with this session representing *the preference for the social novelty phase*. After the termination of the 3CHT test, animals were returned to their home cages in agreement with previously defined housing conditions. The equipment was cleaned with 20% ethanol to eliminate any scent traces from previously used animals to be ready for the next testing.

The behavioral response of the subject rats in the 3CHT was video recorded by the camera, in high enough resolution to render a quality picture for behavioral scoring, and the obtained material was used for subsequent analysis. Considering social motivation theory (Chevallier et al., 2012), which states three components of motivation (social orientation, social seeking and liking, and maintenance), as well as existing findings on the number of approaches and social interaction itself as a reflection of wanting and liking components of social motivation, respectively (Templer et al., 2018), the following parameters were scored: the number of entries and time spent in the sections (to estimate social orientation in general and orientation toward social novelty in particular), the number of approaches to and the time spent with the N1 along with the number of approaches to and the time spent with the O (to estimate wanting and liking aspects of sociability), the number of approaches to and the time spent with the N2 along with the number of approaches to and the time spent with the N1 (to estimate wanting and liking aspects of social novelty preference). A total number of entrances into lateral chambers of the 3CH test was used as a measure of general motor activity. The videos were encrypted, and the experimenters that analyzed the video material and performed behavioral scoring (using a stopwatch) were unaware of the treatment conditions. The identity of the animals according to the type of social environment in which they grew up was disclosed after quantification was completed. The animals were considered to explore the novel rat and the object when they were approaching the cylinders with their nose at a distance of less than approximately 2 cm.

The exclusion criteria were as follows: (1) no approaches to the N1 or O during the sociability phase (as this could lead to the false preference of the unexplored side of the 3CH during the social novelty preference phase) and (2) damaged/lost video material. In the current study, all animals passed criterion 1. However, one video file that addressed the behavior of middle-adolescent animals in the social novelty preference phase of the 3CHT was damaged, so the score of the same animal obtained in early-adolescence, in the same phase of the 3CHT, was excluded as well (to enable a proper analysis of the group tendency across maturation). Due to this exclusion in the results that address *the preference for the social novelty phase*, the number of animals in the GH group was 6 and that in the SH group was 7 (in the results that address *the sociability phase*, the number of animals per group was 7 in both GH and SH groups).

To determine relative preferences, we calculated the discrimination indexes (DI) as ratio scores (Akkerman et al., 2012) by dividing the amount of particular activity in a given compartment [the number of approaches (DIna) and the exploratory time (DIet)] by the total activity during the particular phase (on the same grounds). Thus, for the sociability phase, DIna = number of approaches to N1/number of approaches to N1 + number of approaches to O; DIet = exploratory time of N1/exploratory time of N1 + exploratory time of O. For the social novelty preference phase, DIna = number of approaches to N2/number of approaches to N2 + number of approaches to N1; DIet = exploratory time of N2/exploratory time of N2 + exploratory time of N1. The preference for the N1 section over the O section was calculated through the number of entries in the section (Dines) as the number of entries into the N1 section/number of entries into N1 + number of entries into the O section; the preference for the N2 section over the N1 section was calculated as the number of entries into the N2 section/number of entries into N2 + number of entries into the N1 section. Calculated DIs were, in addition to within- and between-group comparisons, compared to the chance exploration level of 0.5. Such a way of analysis enables control for variability associated with individual differences in the exploration. This way of expressing results is usually used in tests that assess the free choice between two offered items/stimuli regardless of the nature of what is offered (Bevins and Besheer, 2006; Strekalova et al., 2011).

**FIGURE 2:**
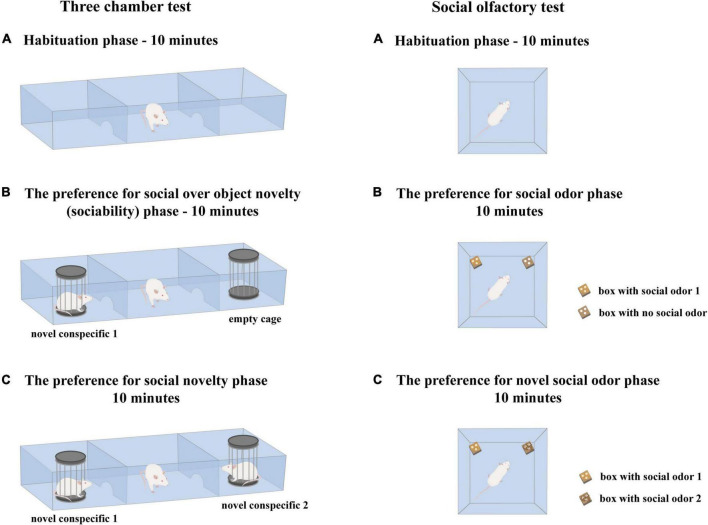
Schematic presentation of experimental procedures related to the 3-chamber test and the social olfactory test. The schemes are given to visualize uniform handling procedures used in the 3-chamber test and the social olfactory test.

The location of the N1 and an empty cage in the left vs. the right side chamber was counterbalanced. The N1 and N2 were the same age and sex as the subject animal. The social behavior of demonstrator rats was not examined. Between phases, the subject animal was returned to the home cage to maximally uniform the handling procedure used in the 3CHT and the social olfactory test used to assess the response of the animals to social odors (schematic presentation of the experimental procedure is given in Figure 2).

#### Experiment 2: Social Olfactory Test: Recognition of a Social Scent and the Preference for the New

The social olfactory test was performed to assess the role of social olfactory cues in the behavior of rats that mature under different housing conditions and to improve the knowledge of the role of social odors in motivated social behavior in rats (Haskal et al., 2020). As adolescence is not a homogenous period and implies going through the maturational phases, the behavior toward social olfactory cues could also be subject to change. In the current study, we used an adaptation of the spontaneous novel object recognition test (Ennaceur and Delacour, 1988; Pavkovic et al., 2018), with the novel odor-marked objects as a modification.

##### Apparatus and Supporting Material

The testing was performed in Opto-Varimex cages (44.2 cm × 43.2 cm × 20 cm), placed in a light- and sound-isolated room provided with indirect and homogenous illumination (150 lx in the center of the arena). Perforated plastic boxes (27 mm × 27 mm × 22 mm, made of transparent material, with three holes in each side and the possibility to open) were used as additional equipment. The bedding material (autoclaved wood shavings) was used for 3 days in additional cages with animals of the same age and sex as the subject rats (the same number of animals per cage), thus containing the smell of their urine, was used as a source of social odors. The bedding material from the home cage of the subject rats was never used. Perforated plastic boxes were filled with the bedding material harvested on the day of testing: it was stored in clearly marked zip-lock bags at room temperature and used within 6 h of sampling to maintain strong and consistent social odor (Arbuckle et al., 2015).

##### Test Procedure

The animals were habituated to the testing room for 1 h. The subject rat was then placed in the open arena (Opto-Varimex cages) and allowed to freely explore the novel environment for 10 min (*habituation phase*). After this session, the subject rat was briefly returned to the home cage, and two perforated boxes [one with the first bedding as a source of social odor 1 (S1) and the other empty, with no social odor (NS)] were placed in the opposite corners of the arena. The subject animal was then gently placed in the center of the arena to freely explore for an additional 10 min, and this session represented *the preference for the social odor phase* (sociability-like phase). Thereafter, the subject rat was briefly returned to the home cage, and the empty perforated box was replaced with the one containing the second bedding material as a source of social odor 2 (S2). Then, the subject rat was gently placed in the center of the arena and allowed to freely explore for the next 10 min, with this session representing *the preference for a novel social odor phase* (social novelty preference-like phase). After the termination of this period, the tested animals were returned to their home cages in agreement with previously defined housing conditions. The equipment was cleaned with 20% ethanol to eliminate any scent traces from previously used animals to be ready for the next testing.

A schematic presentation of the experimental procedure is given in Figure 2. The location of an empty box and social odor 1 box in the left versus the right corner of the cage was systematically alternated between trials. The behavioral response of subject rats during testing was video recorded, and the obtained material was used for subsequent analysis. The following parameters were scored: the number of approaches to and the time spent with the S1 box along with the number of approaches to and the time spent with the NS box (to estimate wanting and liking aspects of social odor preference) and the number of approaches to and the time spent with the S2 box along with the number of approaches to and the time spent with the S1 box (to estimate wanting and liking aspects of the novel social odor preference). The videos were encrypted, and the identity of the animals according to the type of social environment in which they grew up was disclosed after quantification was completed. The animals were considered to explore when they were approaching the boxes with their nose at a distance of less than 2 cm. Behavioral scoring was done with a stopwatch.

To determine relative preferences we calculated the discrimination indexes (DIs) as ratio scores (Akkerman et al., 2012): for the *preference for the social odor phase*, based on the number of approaches (the number of approaches to the S1 box/number of approaches to the S1 box + number of approaches to the NS box); and based on the exploration time (the time spent in the S1 box exploration/time spent in the S1 box exploration + time spent in the NS box exploration): for the *preference for novel social odor phase*, based on the number of approaches (the number of approaches to the S2 box/number of approaches to the S1 box + the number of approaches to the S2 box) and based on the exploration time (the time spent in the S2 box exploration/time spent in the S1 box exploration + time spent in the S2 box exploration). The calculated DIs were, in addition to within- and between-group comparisons, compared to the chance exploration level of 0.5.

The exclusion criteria for the social olfactory test were: (1) no approaches to the S1 or NS boxes during the preference to the social odor phase (as this could lead to the false preference for unexplored part of the testing chamber during the preference for a novel social odor phase) and (2) damaged/lost video material. In the current study, both criteria were met, and there was no exclusion of animals (the number of animals was 8 per group in both GH and SH groups during the entire experimental period).

#### Experiment 3: Immunohistochemical Analysis of the Parvalbumin-Expressing Interneurons in the Hippocampal Subfields

Bearing in mind the role of the hippocampus in the mapping and learning of social space (Leblanc and Ramirez, 2020) and the sensitivity of hippocampal PVIs to SI in the adult rat brain (Filipovic et al., 2013, 2018), an immunohistochemical analysis of the PVIs in the hippocampal subfields was done to assess the relation of findings with the spatial behavior of the animals in the 3CH test, as well as to consolidate age-related outcomes of this adverse social experience.

The animals were decapitated after the first and the second weeks of defined housing. The brains were isolated on ice, fixed in 4% paraformaldehyde for 24 h, and cryopreserved in graded sucrose solutions (10–30% w/v sucrose/PBS). The brains were frozen on isopentane and stored at −80°C until sectioning on a cryotome. The brains were cut in coronal sections 20 μm thick and mounted on slides, dried for 12 h at room temperature, and stored at −20°C until staining.

For further immunohistochemical analysis, the dorsal hippocampus was analyzed, regarding the adult rat atlas brain (−2.9 to −3.6 mm from the bregma, Paxinos and Watson, 2005). Four slices per animal, with the two consecutive analyzed slices being at least four slices apart, were used. Staining for PV and a Purkinje cell protein 4 (PCP4) was used to delineate CA2/CA3 and CA2/CA1 borders, and the PVIs were analyzed in all hippocampal subfields [the pyramidal layer in CA1-3; the granular layer in the dorsal and ventral horn of dentate gyrus (DG) along with the hilar region]. The CA1 was distinguished from the subiculum by the consistency of cell packing in the pyramidal layer, which is more loosely packed in the subiculum than in the CA1 (O’Mara, 2005).

##### Immunofluorescence Staining

Before staining, microscopic slides (previously stored at −20°C) were kept at room temperature (RT) for 1 h. Sections were blocked in 1.5% bovine serum albumin (BSA) in PBS for 30 min at RT and 0.1% Triton X-100 in PBS for 15 min. Sections were then incubated overnight at 4°C with the primary antibody - mouse anti-parvalbumin (P3088, Sigma-Aldrich, St. Louis, MO, United States; 1:2000). The next day, staining was continued with the appropriate secondary antibody (anti-mouse conjugated to Alexa 488, Invitrogen, Carlsbad, CA, United States) used at the 1:300 concentration in PBS for 2 h at RT. Additionally, the same slices were used for CA2 subfield staining with primary antibody - rabbit anti-PCP4 (HPA005792, Sigma-Aldrich, St. Louis, MO, United States; 1:200), followed by the appropriate secondary antibody the next day (anti-rabbit conjugated to Alexa 555, Invitrogen, Carlsbad, CA, United States) used at the 1:250 concentration in PBS for 2 h at RT. For nucleus staining, the Hoechst solution was used (33258, Sigma-Aldrich, St. Louis, MO, United States, 1:1000). Microscopic slides were mounted with Mowiol and left to dry. Samples from different housing (SH vs. GH) and different time points (1 week versus 2 weeks of different housing) were processed in parallel to avoid any non-specific effect of the staining. Sections incubated in parallel without the primary antibody were included as negative controls for autofluorescence and background binding of the secondary antibody.

##### Quantitative Analysis of Parvalbumin Cells in Different Hippocampal Regions

Images were captured on an AxioObserver Microscope Z1 using an AxioVision 4.6 software system (CarlZeiss, Germany) at a magnification of 10×. PV positive cells were counted manually directly under the microscope at a magnification of 20×, and only PV^+^ cells with a visible nucleus were taken into account. The sections were analyzed by a researcher unaware of the experimental condition. Four slices were used per animal, with two consecutive analyzed slices being at least four slices apart. For each selected slice, immunoreactivity was counted per hippocampal subfield (CA1, CA2, CA3, and DG) as the region of interest in both the left and right hemispheres. For each animal/brain, the mean of obtained eight values for each subfield (4 slices × 2 hemispheres) was averaged to obtain a single measure of the number of PVIs in a particular subfield (per section per side per brain).

### Statistical Analysis

The data were presented as means ± *SD*, with individual data plots along the column bars, and were statistically analyzed using Statistica 6 software (StatSoft Inc., Tulsa, OK, United States). The normality of data sets was estimated by Shapiro-Wilk’s test. The accepted level of significance was *p* ≤ 0.05 for all tests. The statistical results along with the tests used, and the exact *p* values are summarized in Supplementary Table S1.

For the data that did not have a normal distribution, non-parametric statistics were used: the Wilcoxon’s test for repeated measures to assess adaptive behavior over time and the Mann–Whitney *U*-test for pairwise comparisons. For the data that passed the normality criteria, the repeated measure ANOVA with the housing condition and maturation (repeated measure) as factors was used, followed by a *post hoc* Tukey test or Unequal N HSD, if applicable.

To estimate the preference, the mean value for each group was compared to the chance levels (0.5) using a one-sample *t*-test.

## Results

### Behavior of the Animals in the 3-Chamber Test – The Preference for Social Over Non-social Context

#### The Number of Entries and the Time Spent in the Section With the Novel Conspecific Versus the Section With the Object (Social Orientation – Preference for Social Space)

**FIGURE 3:**
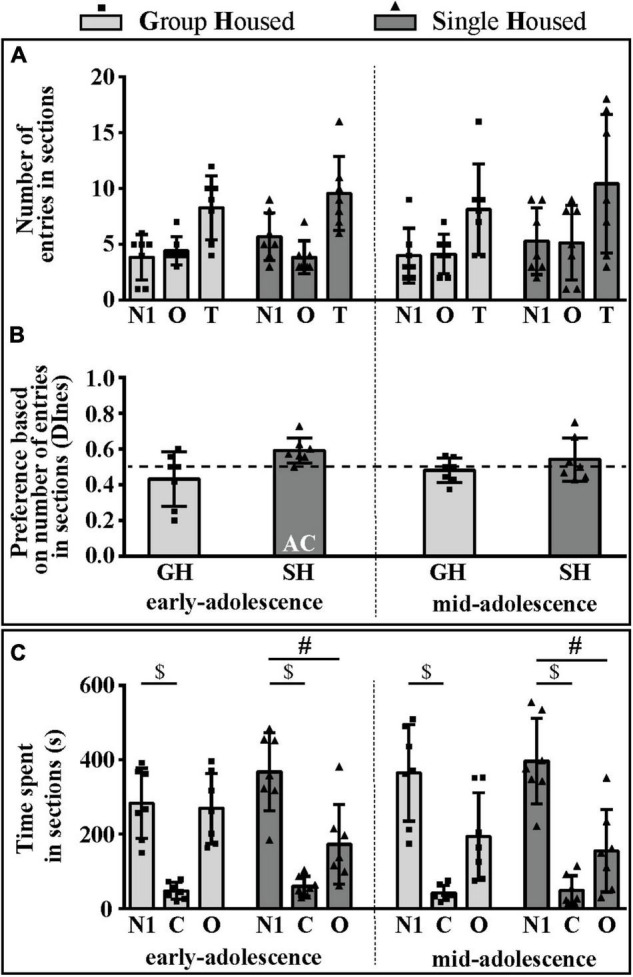
The social orientation of peripubertal male rats in the 3-chamber test at the end of the first week (early-adolescence) and the second week (mid-adolescence) of defined housing, which began on the 29th postnatal day. The data are represented as mean ± *SD*, with individual data plots along the column bars. **(A)** The number of entries into compartments with the social stimulus (novel conspecific – N1) and the empty cup (object – O) and the total number of entries (T). **(B)** Discrimination index based on the number of entries into sections (DInes), addressing “wanting” aspect of the preference for social (N1) section. **(C)** Time spent in N1 section, O section, and central (C) section, reflecting “liking” aspect of the preference for social (N1) section over other two sections; AC – significantly above the chance level (0.5); ^$^*p* ≤ 0.05 (time spent in the N1 section vs. time spent in the C section); ^#^*p* ≤ 0.05 (time spent in the N1 section vs. time spent in the O section). Statistical comparisons and exact *p*-values are given as Supplementary Table S1.

The number of entries into compartments with the social stimulus (novel conspecific – N1, within an inverted wire cup; N1 section) and the empty cup (object – O; O section) was balanced in all examined groups of animals except in rats subjected to SH during early adolescence (Figure 3A). Consequently, relative preference for the social (N1) over the non-social side (O) was shown in the early-adolescent SH group since only in this group the DInes was above the chance (0.5) level (Figure 3B; one-sample *t*-test: *p* = 0.014, *t* = 3.428, df = 6). Of all of the examined parameters [number of entries to the N1 and O sections, total number of entries (T), and the DInes], significant changes during maturation and under different housing conditions were observed for the DInes only, regarding housing conditions [*F*_(1,12)_ = 5.864, *p* = 0.032].

The time spent in the N1 section was higher than the time spent in the center (C) in all examined groups of animals (Figure 3C; $ *p* < 0.001, *t*-test for dependent samples), while compared to the time spent in the O section it was higher only in rats subjected to SH during both early- (Figure 3C; ^#^*p* = 0.049) and early-to-mid-adolescence (^#^*p* = 0.029). Changes in examined parameters (time spent on the N1 side, the non-social/O side, and in the center/C) during maturation and under different housing conditions were not statistically significant.

All statistical details are given as Supplementary Table S1. Overall, these results suggest that, in the sociability phase of testing in the 3CH test, adolescent GH rats do not express a preference for social space either through appetitive or consummatory behavior. On the other hand, SH promotes the preference for social space in such a way that consummatory preference is evident, regardless of animals’ age, but appetitive preference is evident only in early-adolescent SH rats.

#### The Number of Direct Approaches to and the Time Spent in Direct Contact With the Novel Conspecific Versus the Object (Sociability)

**FIGURE 4:**
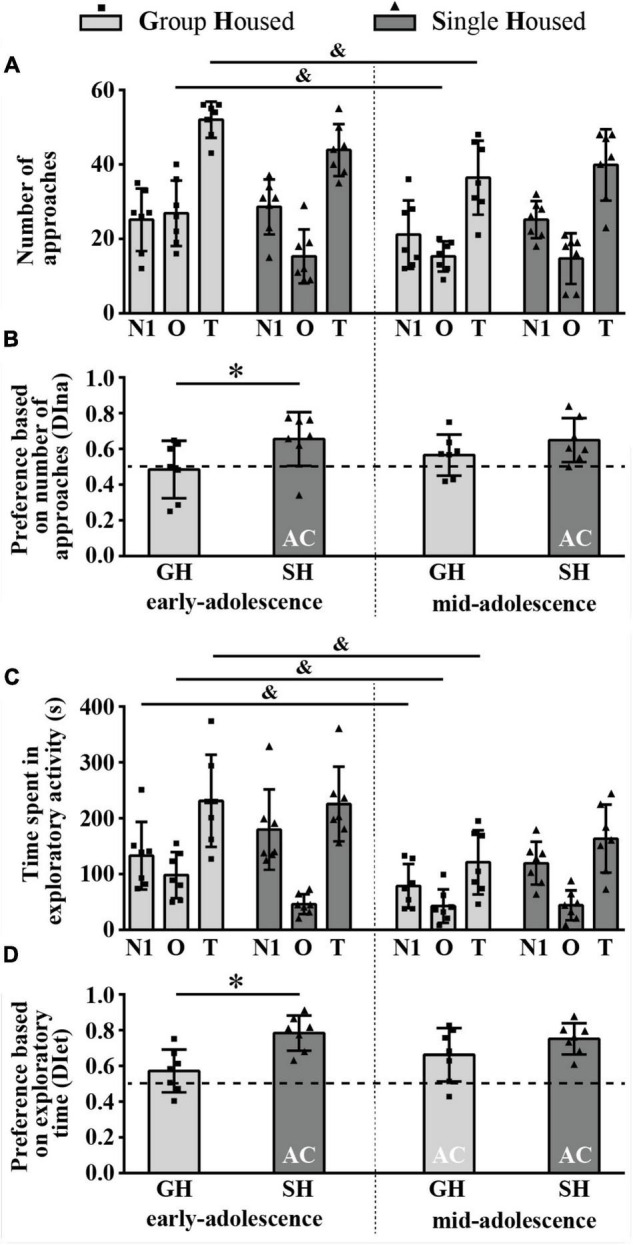
The sociability of peripubertal male rats in the 3-chamber test at the end of the first week (early-adolescence) and the second week (mid-adolescence) of defined housing, which began on the 29th postnatal day. The data are represented as mean ± *SD*, with individual data plots along the column bars. **(A)** The number of approaches to the N1, the O, and the total number of approaches (T). **(B)** Discrimination index based on the number of approaches (DIna), addressing the “wanting” aspect of the preference for the N1 over the O, i.e., sociability. **(C)** Time spent in the exploration of the N1 and the O and total exploratory time. **(D)** Discrimination index based on exploratory time (DIet), addressing “liking” aspect of the preference for the N1 over the O, i.e., sociability. AC – significantly above the chance level (0.5); ^&^*p* ≤ 0.05 (early-adolescence vs. mid-adolescence (maturation) within the same housing conditions); *p ≤ 0.05 (single housed vs. group housed within the same maturational period). Statistical comparisons and exact *p*-values are given as Supplementary Table S1.

The number of approaches to the N1 and the O was balanced in early- and mid-adolescent GH rats but not in SH groups (Figure 4A) and, consequently, the discrimination index based on the number of approaches (DIna) revealed social preference in early- and early-to-mid-adolescent SH groups (Figure 4B, one-sample *t*-test: *p* = 0.035, *t* = 2.716, df = 6 and *p* = 0.014, *t* = 3.428, df = 6, respectively). Factorial analysis revealed (Figure 4A), for the number of approaches to the N1, no significant effects of both factors (housing and maturation) and their interaction; for the number of approaches to the object, a significant effect of maturation [*F*_(1,12)_ = 7.107, *p* = 0.021] and housing × maturation [*F*_(1,12)_ = 5.832, *p* = 0.033], with a decrease in the number of approaches to the object in GH rats across maturation (Figure 4A, ^&^*p* = 0.017, Tukey test); and for the total number of approaches, a significant effect of maturation [*F*_(1,12)_ = 10.6, *p* = 0.007] accompanied by a decrease in the examined parameter in GH rats (Figure 4A, ^&^*p* = 0.015). The DIna (Figure 4B) was significantly higher in early-adolescent SH animals than in GH peers (*p = 0.025, *U* = 7), with no such difference in mid-adolescent animals; the Wilcoxon test did not reveal significant changes in the DIna across maturation within particular housing conditions.

Figure 4C summarizes the results of the time spent in direct exploration of the N1 and O as well as total exploratory time. The analysis of the data revealed that the time spent in the N1 exploration was without significant difference between GH and SH rats of the same age but decreased during maturation in GH rats only (Figure 4C, ^&^*p* = 0.043, Wilcoxon test); the time spent in the O exploration significantly changes during maturation [*F*_(1,12)_ = 18.494, *p* = 0.001]; maturation × housing [*F*_(1,12)_ = 16.008, *p* = 0.002], with a decrease in the examined parameter in GH rats only (Figure 4C, ^&^*p* = 0.001); the total exploratory time significantly changes during maturation [*F*_(1,12)_ = 16.014, *p* = 0.002], with a decrease in examined parameter in GH rats only (Figure 4C, ^&^*p* = 0.016).

Figure 4D summarizes the preference based on the exploratory time (DIet) and represents the relative preference for the N1. There was a social preference (above chance level, 0.5) in mid-adolescent GH animals (Figure 4D; one-sample *t*-test: *p* = 0.028, *t* = 2.876, df = 6) as well as in both early- and early-to-mid adolescent SH groups (*p* < 0.001, *t* = 7.589, df = 6 and *p* < 0.001, *t* = 7.633, df = 6). The two-way ANOVA revealed a significant influence of housing conditions [*F*_(1,12)_ = 11.759, *p* = 0.005] and the *post hoc* analysis showed greater preference in early-adolescent SH rats compared to their GH peers (Figure 4D, *p = 0.023).

All statistical details are given as Supplementary Table S1. Overall, the above-described results show that, in adolescent GH rats, sociability manifests with age in mid-adolescence, strictly in the form of consummatory preference and regardless of the maturation-related decrease in exploratory time. On the other side, SH promotes both appetitive and consummatory aspects of sociability, regardless of animals’ age, which is in comparison to the age-matched GH counterparts evident only in early-adolescent rats.

### Behavior of the Animals in the 3-Chamber Test – The Preference for Social Novelty

#### The Number of Entries and the Time Spent in the Section With a Novel Conspecific Versus the Section With a Familiar Conspecific (Orientation Toward Social Novelty)

**FIGURE 5:**
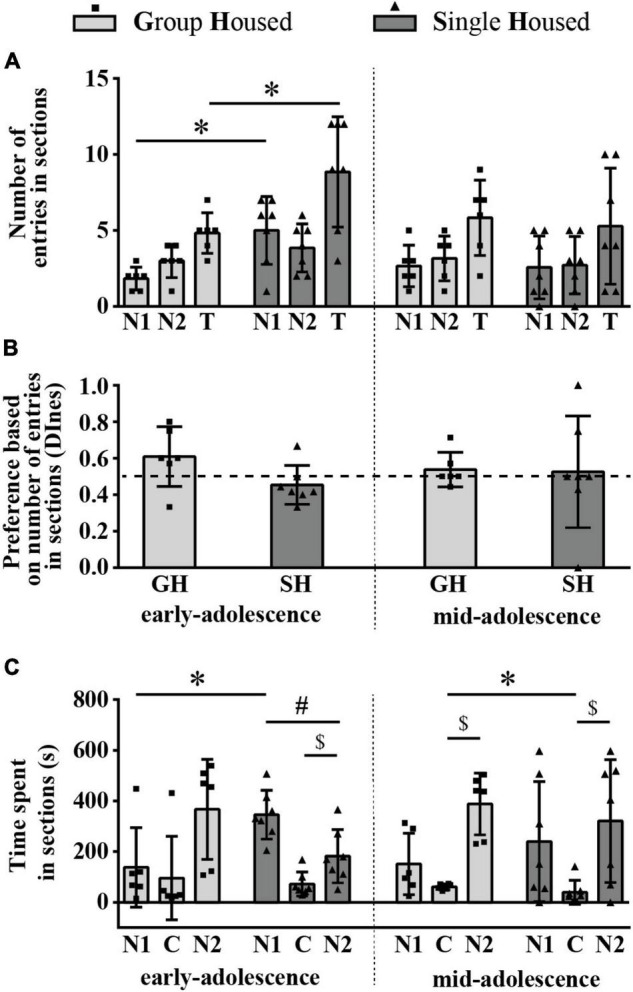
The preference for novel social space in peripubertal male rats at the end of the first week (early-adolescence) and the second week (mid-adolescence) of defined housing, which began on the 29th postnatal day. The parameters were obtained in the 3-chamber test. The data are represented as mean ± *SD*, with individual data plots along the column bars. **(A)** The number of entries into compartments/sections with the familiar conspecific (N1), the novel conspecific (N2), and the total number of entries (T). **(B)** Discrimination index based on the number of entries into sections (DInes), addressing “wanting” aspect of the preference for the section with social novelty (N2 section). **(C)** Time spent in the N1 section, N2 section and central (C) section, reflecting “liking” aspect of the preference for the section with social novelty (N2 section) over other two sections; ^$^*p* ≤ 0.05 (time spent in the N2 section vs. time spent in the C section); ^#^*p* ≤ 0.05 (time spent in the N2 section vs. time spent in the N1 section); *p ≤ 0.05 (single housed vs. group housed within the same maturational period). Statistical comparisons and exact *p*-values are given as Supplementary Table S1.

The number of entries into compartments with a familiar conspecific N1 and the novel conspecific (N2) was balanced in all examined groups of animals (Figure 5A) and the discrimination index based on the number of entries into sections (DInes) showed no preference for the N2 side in all examined groups of animals (Figure 5B). Two-way ANOVA revealed a significant effect of housing conditions on the number of entries into the N1 side and on the total number of entries [*F*_(1,11)_ = 14.442, *p* = 0.003, and *F*_(1,11)_ = 5.788, *p* = 0.035, respectively], and a *post hoc* analysis revealed a significant increase in both examined parameters in early-adolescent SH compared to early-adolescent GH rats (Figure 5A; *p = 0.001 and *p = 0.014, respectively, HSD test for unequal samples). There were no significant effects of maturation, housing conditions, and their interaction on the DInes (Figure 5B). Overall, these findings indicate that the presence of social novelty produced hyperactivity in early-adolescent SH rats compared to their GH counterparts, promoting entrances into the section with the familiar animal.

Figure 5C summarizes the results of the time spent in the sections. The time spent in the N2 section was significantly higher than the time spent in the center (C) in all (0 < *T* < 2, 2.028 < *Z* < 2.201, 0.028 < $ *p* < 0.043, Wilcoxon test) except in the early-adolescent GH group (*T* = 2, *Z* = 1.782, *p* = 0.075). Compared to the time spent in the N1 section, the time spent in the N2 section was different/lower only in early-adolescent SH rats (Figure 5C; *T* = 2, *Z* = 2.028, ^#^*p* = 0.043), indicating social orientation toward the space with a familiar subject in this experimental group. Changes in examined parameters (time spent in the N1, N2, and C sections) during maturation were not statistically significant neither in GH nor in the SH group, while housing conditions during a particular period of maturation showed an effect on the time spent in the N1 section in early-adolescent animals (Figure 5C; through an increase in SH compared to GH group, *p = 0.032) and in mid-adolescent animals concerning the time spent in the center (Figure 5C; through a decrease in SH compared to GH group, *p = 0.038).

All statistical details are given as Supplementary Table S1. Overall, the above-described results of the 3CH test demonstrate that, in maturing rats, neither of the tested housing conditions (GH, SH) produces a preference for space with a social novelty. Moreover, early-adolescent SH animals showed an increased number of entries into a familiar chamber (as well as a total number of entries) compared to their GH counterparts and stand as the only group with a consummatory preference for a familiar social space.

#### The Number of Direct Approaches to and the Time Spent in Direct Contact With the Novel Versus an Old Conspecific (Social Novelty Preference)

**FIGURE 6:**
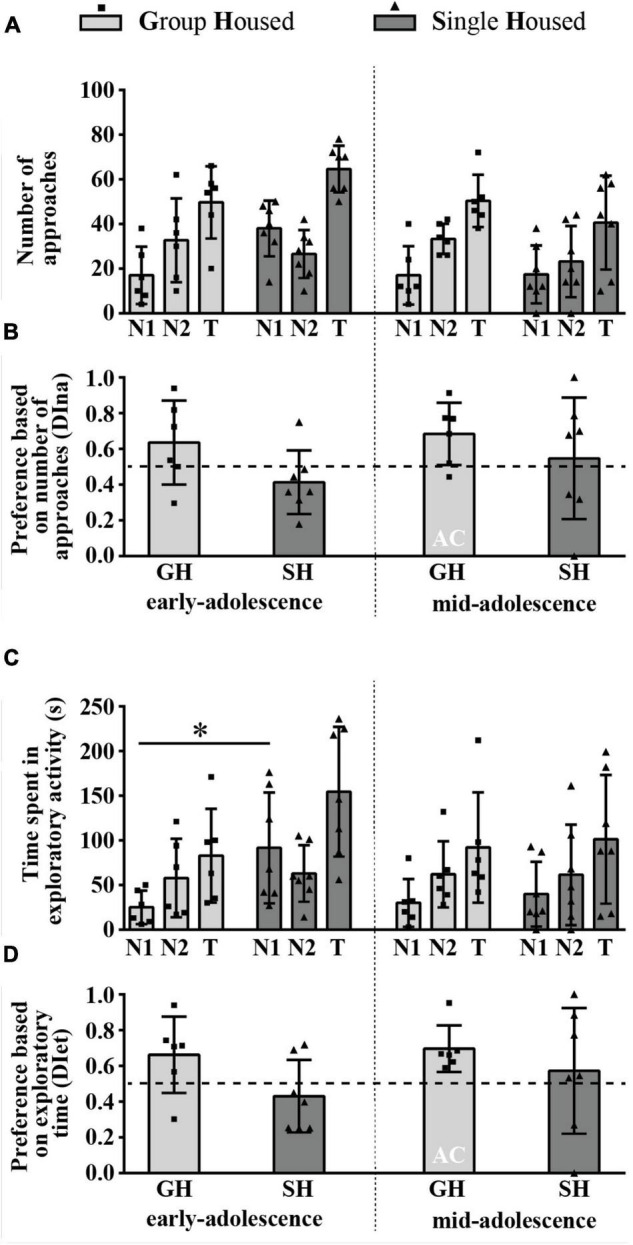
Social novelty preference of peripubertal male rats in the 3-chamber test at the end of the first week (early-adolescence) and the second week (mid-adolescence) of defined housing, which began on the 29th postnatal day. The data are represented as mean ± SD, with individual data plots along the column bars. **(A)** The number of approaches to the N1 and the N2 and the total number of approaches (T); **(B)** Discrimination index based on the number of approaches (DIna), addressing the “wanting” aspect of the preference for the N2 over the N1, i.e., social novelty preference; **(C)** Time spent in the exploration of the N1 and the N2 and the total exploratory time; **(D)** Discrimination index based on exploratory time (DIet), addressing “liking” aspect of the preference for the N2 over the N1, i.e., social novelty preference. AC – significantly above the chance level (0.5); *p ≤ 0.05 (single housed vs. group housed within the same maturational period). Statistical comparisons and exact *p*-values are given as Supplementary Table S1.

Figure 6A summarizes the results of the number of direct approaches to the familiar (N1) and the novel (N2) animal as well as the total number of approaches (T), while Figure 6B represents the relative preference for the N2 based on the number of approaches (DIna). The two-way ANOVA did not reveal a significant influence of housing conditions, maturation, and their interaction on all examined parameters. The preference for social novelty based on the DIna was observed only in mid-adolescent GH animals as, only in this group, the examined parameter was above the chance level (0.5) (Figure 6B; one-sample *t*-test, *p* = 0.048, *t* = 2.590, df = 5).

Figure 6C summarizes the results of the time spent in the exploration of the familiar (N1) and the novel (N2) animal as well as the total exploratory time (T), while Figure 6D represents the relative preference for the N2 based on the exploratory time (DIet). Two-way ANOVA showed a significant effect of housing conditions on the time spent in exploration of the N1 [Figure 6C; *F*_(1,11)_ = 9.59, *p* = 0.011], and a *post hoc* analysis revealed that early-adolescent SH rats spent more time in the N1 exploration than their GH counterparts (Figure 6C; *p = 0.017, Tukey test for unequal samples). For the other three examined parameters (N2 exploration, T, and the DIet), two-way ANOVA did not reveal significant effects of housing conditions, maturation, and their interaction. The preference for social novelty based on the DIet was detected in mid-adolescent GH animals (Figure 6D; one-sample *t*-test, *p* = 0.014, *t* = 3.693, df = 5).

All statistical details are given as Supplementary Table S1. Overall, the results obtained in the 3CH test show that, in adolescent GH rats, social novelty preference appears with age, in mid-adolescence, through both appetitive and consummatory aspects of social motivation. SH abolishes preference for social novelty in mid-adolescent rats, while in early-adolescent rats, regardless of promoting the preference for familiar social space in these animals (as described in the previous section), it does not lead to a preference for either the familiar or the novel conspecific (prioritized memory for social space with a familiar animal but not for the animal as such).

### Behavior of the Animals in a Social Olfactory Test – The Preference for Social Odor Phase

**FIGURE 7:**
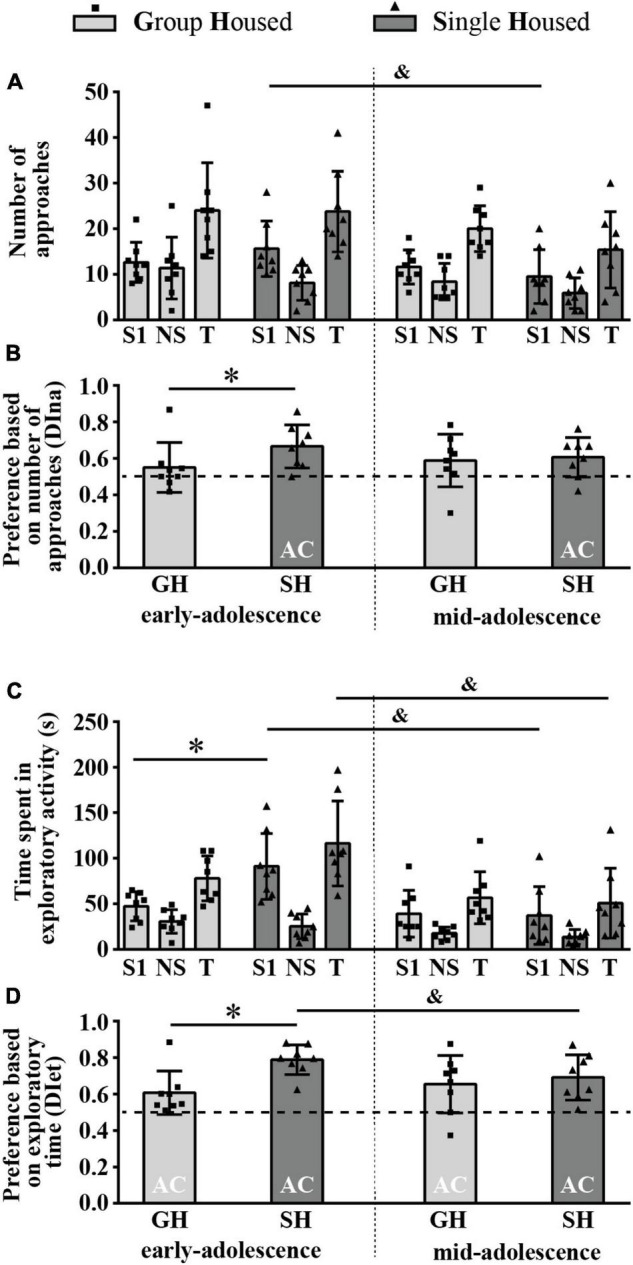
The behavior of peripubertal male rats in a social olfactory test (the preference for social odor phase) at the end of the first week (early-adolescence) and the second week (mid-adolescence) of defined housing, which began on the 29th postnatal day. The data are represented as mean ± *SD*, with individual data plots along the column bars. **(A)** The number of approaches to the box with the novel social odor (S1) and to the box with no smell (NS) and the total number of approaches (T). **(B)** The relative preference for the S1 is based on the number of approaches to the boxes. **(C)** The time spent in the exploration of the S1 box and the NS box and the total exploratory time (T). **(D)** The relative preference for the S1 is based on exploratory time. AC – significantly above the chance level (0.5); ^&^*p* ≤ 0.05 (early-adolescence vs. mid-adolescence (maturation) within the same housing conditions); *p ≤ 0.05 (single housed vs. group housed within the same maturational period). Statistical comparisons and exact *p*-values are given as Supplementary Table S1.

Figure 7A summarizes the results of the number of approaches to the box with social odor 1 (S1) and the box with no smell (NS) and the total number of approaches (T). The two-way ANOVA revealed a significant effect of maturation on the number of approaches to the S1 [*F*_(1,14)_ = 6.723, *p* = 0.021], and a *post hoc* analysis revealed a significant decrease in this parameter in SH (but not in GH) rats across maturation (Figure 7A; ^&^*p* = 0.032, Tukey test). Maturation appeared as a significant factor for the total number of approaches (T) as well [*F*_(1,14)_ = 4.718, *p* = 0.048]. Neither maturation nor housing conditions influenced the number of approaches to the NS.

Figure 7B represents a relative preference for the S1 based on the number of approaches to the boxes, assessed by the discrimination index (DI). Preference for the S1 box (the DI above chance level, 0.5) was detected in SH animals regardless of their age (Figure 7B; one-sample *t*-test: early-adolescence *p* = 0.005, *t* = 3.979, df = 7 and mid-adolescence *p* = 0.029, *t* = 2.751, df = 7). It was significantly higher in early-adolescent SH animals than in the GH peers (Figure 7B; *p = 0.046, *U-*test), with no difference in mid-adolescent animals; the Wilcoxon test did not reveal significant changes in this parameter across maturation within particular housing conditions.

Figure 7C summarizes the results of the time spent in the exploration of the box with social odor 1 (S1) and the box with no smell (NS) and the total exploratory time (T). Two-way ANOVA revealed a significant effect of maturation and maturation × housing conditions interaction on the time spent in the S1 exploration [*F*_(1,14)_ = 10.86, *p* = 0.005 and *F*_(1,14)_ = 5.913, *p* = 0.029, respectively], and a *post hoc* analysis revealed a significant increase in this parameter in early-adolescent SH compared to early-adolescent GH rats (Figure 7C; *p = 0.049, Tukey test), as well as a significant decrease in this parameter in SH rats across maturation (Figure 7C; ^&^*p* = 0.006). A significant effect of maturation on the time spent in the NS exploration and on total exploratory time was revealed as well [Figure 7C; *F*_(1,14)_ = 10.971, *p* = 0.005 and *F*_(1,14)_ = 15.381, *p* = 0.002], with a decrease in total exploratory time in SH animals (Figure 7C; ^&^*p* = 0.005).

Figure 7D shows that, based on the time devoted to the S1 and NS exploration, all groups showed preference for the box with social odor (one sample *t*-test: 0.040 < *p* < 0.001, 9.968 < *t* < 2.522, df = 7). The preference was higher in early-adolescent SH compared to early-adolescent GH rats (Figure 7D; *p = 0.016, *U*-test) and slightly but significantly decreased across maturation in SH animals (Figure 7D; ^&^*p* = 0.036, Wilcoxon test).

All statistical details are given as Supplementary Table S1. Overall, the above-described findings show that the consummatory aspect of preference for the social odor is present in maturing rats regardless of the housing conditions (GH, SH), while the appetitive aspect appears only in SH rats regardless of their age. Both aspects of preference for social odor are higher in early-adolescent SH rats than in age-matched GH counterparts. Moreover, in SH rats, overall decrease in interest in the social odor was observed during maturation, which contributed to a decrease in consummatory preference in mid-adolescent compared to early-adolescent rats.

### Behavior of the Animals in a Social Olfactory Test – The Preference for Novel Social Odor Phase

**FIGURE 8:**
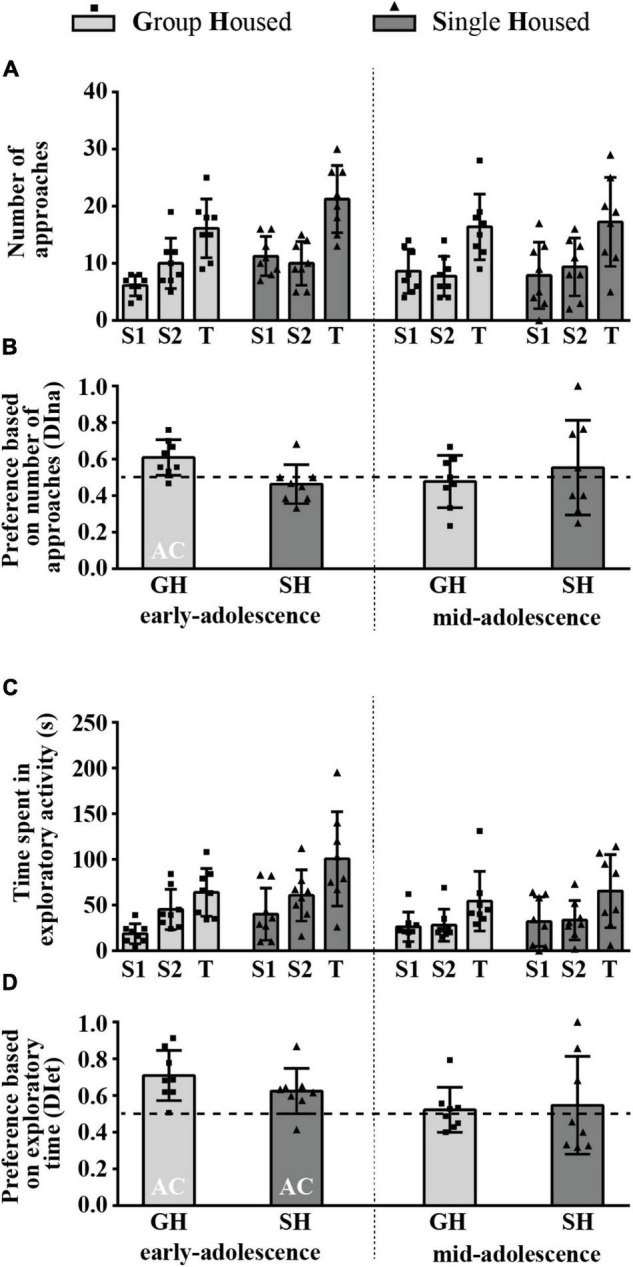
The behavior of peripubertal male rats in a social olfactory test (the preference for novel social odor phase) at the end of the first week (early-adolescence) and the second week (mid-adolescence) of defined housing, which began on the 29th postnatal day. The data are represented as mean ± *SD*, with individual data plots along the column bars. **(A)** The number of approaches to the box with the familiar social odor (S1) and to the box with novel social odor (S2) and the total number of approaches (T). **(B)** The relative preference for the S2 is based on the number of approaches to the boxes. **(C)** The time spent in the exploration of the S1 box, the S2 box, and the total exploratory time (T). **(D)** The relative preference for the S2 is based on exploratory time. AC – significantly above the chance level (0.5). Statistical comparisons and exact *p*-values are given as Supplementary Table S1.

Figure 8A summarizes the results of the number of approaches to the box with the familiar social odor 1 (S1) and the box with novel social odor (S2) and the total number of approaches (T), while Figure 8B represents the relative preference for the S2 based on the number of approaches to the boxes. The two-way ANOVA revealed no significant effect of maturation, housing conditions, and their interaction on any of the examined parameters. The DI was above chance level (0.5) only in early-adolescent GH animals (Figure 8B; one-sample *t*-test: *p* = 0.016, *t* = 3.146, df = 7).

Figure 8C summarizes the results of the time spent in the exploration of the box with the familiar social odor 1 (S1) and the box with the novel social odor (S2) and the total exploratory time (T), while Figure 8D represents the relative preference for the S2 based on exploratory time. There was no significant effect of maturation, housing conditions, and their interaction on any of the examined parameters, except maturation regarding the DI [*F*_(1,14)_ = 5.497, *p* = 0.034]. The DI was above chance level (0.5) in early-adolescent animals regardless of their housing conditions, as revealed by one-sample *t*-test (Figure 8D; for GH group *p* = 0.003, *t* = 4.346, df = 7; for SH group *p* = 0.025, *t* = 2.824, df = 7).

All statistical details are given as Supplementary Table S1. Overall, these data reveal that, in GH rats, the preference for the novel social odor is age-related and exists only in early-adolescent animals. However, while the consummatory aspect of preference is not sensitive to housing conditions, the appetitive aspect is abolished by SH.

### The Number of Neurons With Parvalbumin Immunoreactivity in the Dorsal Hippocampus of Single- and Group-Housed Peripubertal Rats

**FIGURE 9:**
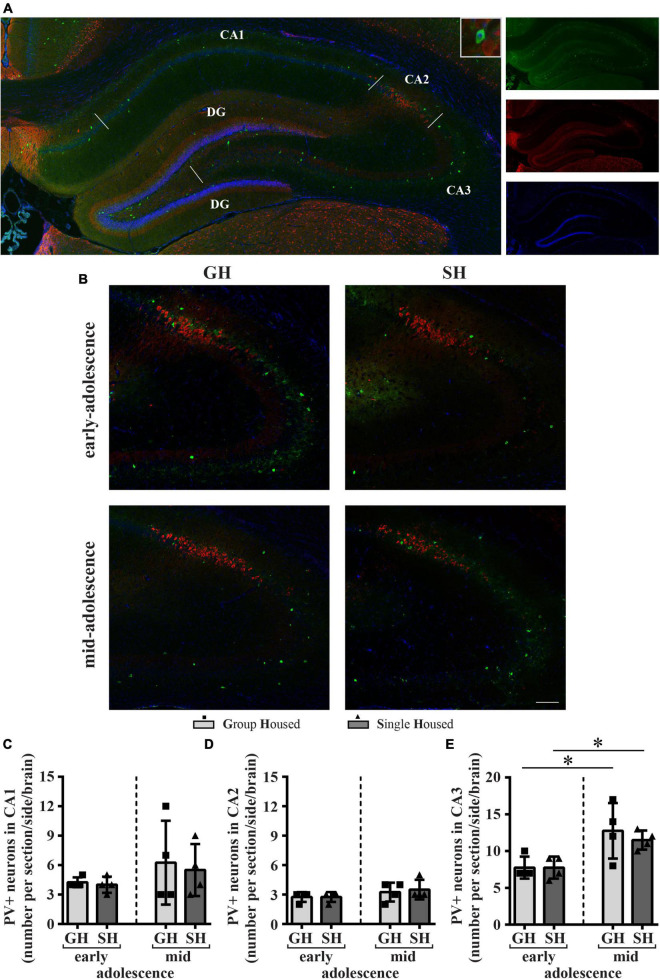
The number of neurons with parvalbumin (PV) immunoreactivity (PVI) in the dorsal hippocampus (the CA1, CA2, and CA3 subfields) of peripubertal rats at the end of the first week (early-adolescence) and the second week (mid-adolescence) of defined housing, which began on the 29th postnatal day. **(A)** A representative panoramic image of the PVI in the hippocampus of a peripubertal rat. **(B)** Representative panoramic images for CA2/CA3 subfields. **(C–E)** The number of PVI-labeled neurons in the CA1, CA2, and CA3 subfields. The dorsal hippocampus was analyzed regarding the adult rat atlas brain (–2.9 to –3.6 mm from the bregma, Paxinos and Watson, 2005). Scale bar 100 μm. *p ≤ 0.05 (early-adolescence vs. mid-adolescence (maturation) within the same housing conditions). Statistical comparisons and exact *p*-values are given as Supplementary Table S1.

**FIGURE 10:**
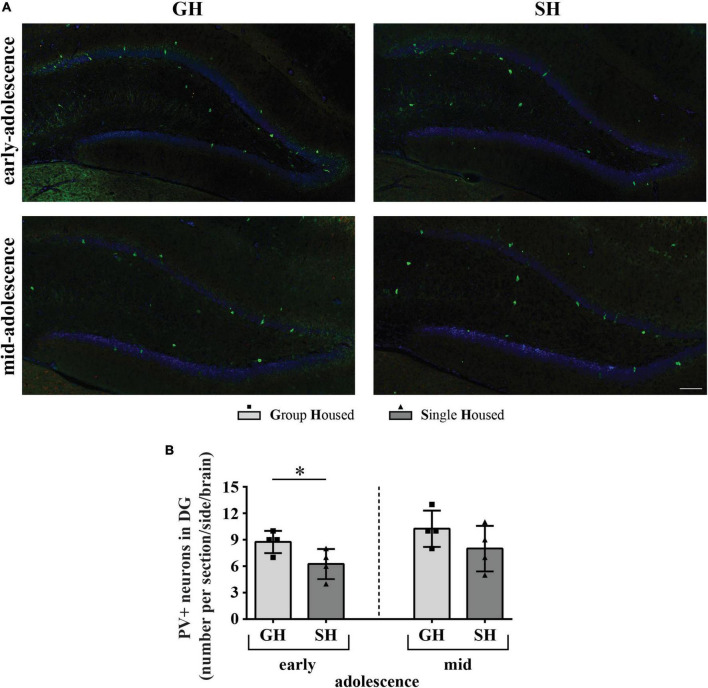
The number of neurons with parvalbumin (PV) immunoreactivity (PVI) in the dentate gyrus of peripubertal rats at the end of the first week (early-adolescence) and the second week (mid-adolescence) of defined housing, which began on the 29th postnatal day. **(A)** Representative panoramic images for the DG. **(B)** The number of PVI-labeled neurons in the DG subfield. The dorsal hippocampus was analyzed regarding the adult rat atlas brain (–2.9 to –3.6 mm from the bregma, Paxinos and Watson, 2005). Scale bar 100 μm. *p ≤ 0.05 (single housed vs. group housed within the same maturational period). Statistical comparisons and exact *p*-values are given as Supplementary Table S1.

A representative panoramic image of the PVI-labeled neurons in the hippocampus of a peripubertal rat is presented in Figure 9A. Separate representative panoramic images are created for CA2/CA3 (Figure 9B) and dentate gyrus (Figure 10A) of the early- and mid-adolescent GH and SH rats.

The number of PVI-labeled neurons in the dorsal CA1, CA2, and CA3 subfields of the hippocampus showed no significant difference between SH and GH rats within a particular period of adolescence (Figures 9C–E). Importantly, housing conditions did not have an influence on the increase in the number of PVI neurons in the CA3 subfield during maturation (Figure 9E; GH regimen *p = 0.043, SH regimen *p = 0.021, *U*-test).

The number of PVI-labeled neurons in the DG was significantly lower in early-adolescent SH compared to early-adolescent GH rats (Figure 10B; *p = 0.043), without much difference between mid-adolescent GH and SH animals.

All statistical details are given as Supplementary Table S1.

## Discussion

Social behavior is composed of a range of highly specific behaviors and there is a great interest in understanding how social experience affects social motivation and cognition and how motivation biases cognition. Using the rat model, this study revealed for the first time the peculiarities in the development of appetitive and consummatory components of motivated social behavior in adolescence. It pays attention to the power of reduced direct contact with peers to affect motivated social behavior at different functional levels and, by promoting an appetitive approach toward peers, to harm the interest for social novelty. This study also revealed that motivation to visit or stay in a social space is not a surrogate measure of sociability/social novelty preference as well as that the response to social odors should not be used as a surrogate measure of sociability and social recognition memory in rodents, thus highlighting the importance of a correct methodological approach when working on the decoding of neurobehavioral consequences of unmet social needs.

### Motivated Social Behavior of Group-Housed Adolescent Rats in the 3-Chamber Test – Social Orientation, Sociability, and Social Novelty Preference

Although it is well known that between adolescence and adulthood the brain undergoes maturation and refinement of synaptic and neural circuits, there are few rigorous studies focusing on behavioral markers of neurodevelopmental trajectories in rodents (Choy et al., 2021). Here, we show that, in typically developing early-to-mid-adolescent GH rats, *social orientation* (preference for social space) is absent while *sociability* (preference for social over object novelty) becomes evident at the end of mid-adolescence, strictly through participation in social contact (consummatory/liking) and not through the appetitive/wanting response. Importantly, exploratory activity devoted to both a peer and an object decreased across early-to-mid adolescence indicating that, in this rat model of adolescent maturation, the *consummatory/liking aspect of sociability appears like the function of adolescent age but not as a directly proportional parameter to the duration of the exploration*. Essentially, the consummatory/liking component of sociability observed at the end of mid-adolescence in GH rats was completely sufficient for the formation of memory on a met peer and the attention allocation to the next social novelty, which was indicated by the fact that social novelty preference was evident in mid-adolescent GH animals through both the appetitive/wanting (number of approaches) and consummatory/liking (direct exploration) components of the motivational response but still without preference for the social space (i.e., social orientation). *These findings, observed in the rodent model of adolescence, for the first time reveal that social orientation (in terms of preference for social space) and the appetitive/wanting component of social motivation in the sociability phase are not characteristics of motivated social behavior in rats that mature in standard living conditions.*

By showing that the consummatory aspect of sociability in rats that mature in standard living conditions is developmentally regulated, this study improves upon the current knowledge that largely implicates the preference for a social opportunity as the expected outcome when examining sociability in normal rodents (Moy et al., 2004; Dai et al., 2018; Templer et al., 2018). In some studies, Wistar rats aged 35 to 40 days represented one experimental group, without specifying the share of the “older” animals in the given group (Dai et al., 2018). However, the reproducibility of our results remains to be verified through other studies that would use the same experimental design and age range, especially given the new findings that point to strain-specific differences in social investigation behavior in rodent models (Netser et al., 2020).

### Motivated Social Behavior of Single-Housed Adolescent Rats in the 3-Chamber Test – Social Orientation, Sociability, and Social Novelty Preference

Our study showed that SH promotes a preference for social space in such a way that consummatory preference is evident regardless of animals’ age, but appetitive preference is evident only in early-adolescent SH rats. Moreover, SH produced both appetitive/wanting and consummatory/liking components of sociability in isolated animals regardless of their age. These data demonstrate that an appetitive approach toward social space is not a prerequisite for the behavior of the animal within it and, therefore, the parameters of a chamber- and a social cue-related activity of the subject animal in the 3CH test should not be equated.

Increased appetitive motivation for a social space during the sociability phase of testing in the 3CH test, observed only in early-adolescent SH rats, could be viewed as a part of active learning about the position of a social space that holds valuable/rewarding content for these animals – a companion. Such behavior is not due to a general increase in locomotor activity in early-adolescent SH rats, since the number of approaches to the social and non-social chamber observed in these animals was highly similar to that observed in others. The fact that early-adolescent SH rats appetitively learn about the position of the social within the physical space is confirmed by the findings that, during the social novelty preference phase, these animals show a preference for the social space with a familiar animal but not for a familiar conspecific (discussed below). Social proximity and play have been used as an incentive for place conditioning experiments in adolescent rodents, using place conditioning, operant lever-pressing, and T-maze discrimination tasks, which suggest a rewarding nature of social interactions in laboratory animals (Trezza et al., 2011). As rewards have been shown to bias attention allocation (Madan, 2017), we propose that a novel conspecific (N1) acquires reward value in early-adolescent SH rats (by stimulating their “liking” response along with “wanting” during the initial encounter/sociability test), thus attracting attention (rewards that are “liked” are usually “wanted”; Berridge et al., 2009) and *shaping socio-spatial cognition.* Notably, by showing that social communication that enables maturing rats restricted (i.e., not free) interaction with a conspecific of the same age and sex is a landmark for early- but not for mid-adolescent SH rats, this study sheds light on the complexity of the rewarding properties of social interaction in adolescent rodents. One of the explanations for this could be that *older animals are more prone to direct social contact, so the restricted ones are not a sufficient source of satisfaction for mid-adolescent SH animals to the extent necessary to give them the status of a landmark.* Play fighting, in which male rats engage more frequently than female rats, reaches its peak frequency between 30 and 40 days of age and is, in the absence of defense by the recipient rat, replaced by other forms of social contact, such as anogenital investigation and climbing over the partner (Pellis and McKenna, 1995; Pellis et al., 1997). It has been proposed that social isolation increases the animals’ emotional reactivity, thus producing more complex cognitive consequences, compared to the lack of play fighting experience that may produce deficiencies in social-cognitive tasks without any increase in anxiety and fear (Schneider et al., 2016; Pellis and Pellis, 2017). Nevertheless, our data did not reveal significant differences in the sociability of early-adolescent SH rats and mid-adolescent SH rats regarding both appetitive (wanting; the number of approaches) and consummatory (liking; exploratory time) aspects of sociability; therefore, diminished motivation for social contact (as a reflection of social anxiety) could not be viewed as the cause of the lack of appetitive preference for social space in mid-adolescent SH rats. It is more likely that, in addition to the two examined behavioral indicators of social motivation, there is a third that speaks much more convincingly about the act of social affiliation in early-adolescent SH rats, and so perhaps, quantifying direct contact (touch) as a parameter can fill in the missing pieces of this puzzle (Ellingsen et al., 2015).

The result that SH produces both appetitive/wanting and consummatory/liking components of sociability in isolated adolescent rats regardless of their age is partly in line with the findings of a study conducted on adult rats in the 3CH test, which shows that, regardless of their living conditions, animals prefer to interact with an unknown conspecific rather than be alone, but that non-socially housed rats engage with an unfamiliar rat more than socially housed rats (Templer et al., 2018). However, in the present study, only early-adolescent SH rats were assessed as more sociable than their GH counterparts, which did not show any sociability marks. *Thus, the finding that objective social isolation leads to an increase in social motivation in rodents should not be generalized, since in maturing animals, it actually promotes the premature appearance of either sociability as a whole (in early-adolescence) or of some of its aspects (the wanting aspect in mid-adolescence, since consummatory preference is confirmed and not improved by SH).* The difference in data presentation in these two studies makes direct comparisons difficult, but it is evident that the increase in sociability in early-adolescent SH rats compared to their GH peers is not observable through the exploratory time, leaving open the question of the degree of similarity of the findings.

While it is well known that motivation can guide cognition, the nuances of these motivation-cognition interactions have to be better understood (Madan, 2017). Our study made its contribution to this topic by showing that, in animals that mature, social isolation does not promote social novelty preference but instead abolishes it (in those maturing animals where it otherwise exists, i.e., in mid-adolescent rats). This is contrary to the results observed in adult rats exposed to non-social housing, based on which the authors concluded that the lack of social interaction in non-socially housed rats may have more impact on social motivation than the consistent opportunity for social contact provided in social living conditions (Templer et al., 2018). Moreover, in our study, SH, as a factor, showed a strong tendency to negatively influence *the consummatory* aspect of social novelty preference (*p* = 0.053) in maturing rats, i.e., worked in favor of *avoidance of social novelty*. Thus, by revealing that *the motivation for social novelty in adolescent rats depends on the satisfied needs for familiarity*, our study sheds light on *the order of the primacy of social needs in maturing rodents*, which is of high importance for a better understanding of the basic biological principles related to social novelty preference in social mammals in general.

### Motivated Behavior of Group- and Single-Housed Adolescent Rats to Social Odors as Cues: Comparison With the Results of the 3-Chamber Test

Our findings have revealed that the detection of social odor and the preference for it in a “liking” manner is present in both age groups of peripubertal GH rats, which indicates that *the absence of sociability in early-adolescent GH rats is not due to a deficit in sensory processing but to general curiosity*. Moreover, early-adolescent rats showed complete (wanting and liking) preference for the novel social odor but not for the novel animal, while mid-adolescent GH animals regardless of the absence of preference for the novel social odor preferred the novel animal – a clear indication that *maturation of social cognition is not simply based on ability to discriminate odors.* Our findings are in agreement with the findings of Professor Wagner’s group that social recognition relies upon the integration of olfactory, auditory, and somatosensory cues, hence requiring an active behavior of social stimuli (Haskal et al., 2020), further extending the knowledge by adding that *this integration, as the base of social cognition, seems to become possible at the end of mid-adolescence*. The autograph of SI in the social odor’s detection/discrimination paradigm was evident through the endorsement of the wanting/appetitive (in addition to consummatory) interest for social odor regardless of the animal’s age and the abolition of appetitive response to a novel social odor in early-adolescent rats. Thus, in agreement with the findings discussed in the subsections above, *unsatisfied social needs principally manipulate the appetitive component of approach behavior in response to social odors, favoring and grounding the creation of a sense of familiarity.* Moreover, the comparison of the response to social odors and live animals shows that *the response to social odors should not be used as an indication of sociability and social recognition memory* (as used in some studies, Jones et al., 2019) because they can lead to a wrong conclusion.

### Changes in the Number of Hippocampal PV^+^ Interneurons Due to Social Isolation in Adolescence – A Potential Link With Appetitive Learning of a Social Space

Our study showed that the hippocampal PV^+^ interneurons, described as highly sensitive to SI in adult male rats (Filipovic et al., 2013, 2018), in maturing rats express age- and subfield-specific sensitivity to housing conditions since a decrease in the number of hippocampal PV^+^ interneurons is detected only in early-adolescent SH rats and only in the DG subfield. Observed results should not be a consequence of increased stress response to SI since the basal level of corticosterone was control-like in early-adolescent SH animals (Potrebic et al., 2021). In the available literature, the influence of SI on the PVIs in maturing rodents has been mainly focused on the medial prefrontal cortex (mPFC) as a part of a network that regulates social behavior, based on findings obtained in socially interacting animals that did not have to previously find/locate a conspecific in the experimental space (Kingsbury et al., 2019; Bicks et al., 2020). According to literature, functions that have been proposed for the DG include pattern separation, pattern completion, novelty detection, and working memory (Hainmueller and Bartos, 2020) as well as the binding of different types of incoming sensory information to spatial contexts (Lee and Jung, 2017). The PV^+^ interneurons in the DG have a central role in suppressing the response of dentate granule cells to excitatory inputs from the entorhinal cortex (Lee et al., 2016) and, through a powerful lateral-inhibition microcircuit, enable the DG to perform efficient pattern separation (Espinoza et al., 2018). From the functional point of view, a reduction in the number of PV^+^ immunoreactive neurons should influence network properties by shifting excitation/inhibition balance toward enhanced inhibition (Schwaller, 2012; Filice et al., 2016), thus contributing to the selective processing of spatially based information. As in the 3CH test, the only group that showed *appetitive learning of social space* was the same one that showed a decrease in the PV^+^ interneurons in the DG, we suppose that this decrease is a part of the neuromodulatory mechanism underlying biased memory computation, which “helps” motivation to influence cognition (Kennedy and Shapiro, 2009; Madan, 2017; Kassab and Alexandre, 2018). Indeed, novel findings indicate an important role of the DG in the mediation of brain responsiveness to rewarding stimuli, by showing that midbrain dopaminergic input in the DG induces long-term depression of cortical inputs and impairs subsequent contextual learning (Du et al., 2016). Although this segment of research requires additional research, the findings speak in favor of the importance of social experience for PV^+^ neurons in the maturing adolescent brain, identifying early-adolescence as a particularly sensitive period and adding the DG to the list of brain regions that sense social experience.

Our study revealed for the first time that the number of PV^+^ expressing interneurons increases in the CA3 of the dorsal hippocampus during early-to-mid adolescence and that this increase is not sensitive to housing conditions. The upregulation of PV^+^ expression in multiple brain regions at specific time points has been described as a part of the developmental program (de Lecea et al., 1995; Hof et al., 1999), and novel findings indicate that PV expression also increases during adolescence in both the mPFC and the ventral hippocampus of rats (Caballero et al., 2013, 2014). The findings of the present study are generally in the line with the literature highlighting protracted maturation of the hippocampal subfields (Keresztes et al., 2018).

### Comparison With Findings in Human Adolescents

As already mentioned, adolescence is a highly conserved developmental stage common among mammalian species (Spear, 2004) and there is a view that rodent stages of adolescence [early adolescence (P21–34), mid-adolescence (P34–46), and late adolescence (P46–59)] roughly correspond to human adolescence [early (10–14 years), middle (15–17 years), and late (18–21 years)] (Burke and Miczek, 2014).

Our findings indicate that *the presence of peers in early adolescence is like a basic physiological need*, so the preference for the social context does not manifest unless this basic need is endangered, while in mid-adolescence, the liking aspect of sociability naturally appears along with social recognition, demonstrating that *mid-adolescence is the period when the individual attitude toward the society begins to change*. The results of the present study are generally in agreement with the findings in humans, which state that social recognition, identity perception, and probably social cognition, in general, take place in mid-adolescence (Fuhrmann et al., 2016). Also, humans generally become more extraverted and more emotionally stable during maturation, thus becoming increasingly ready to socially invest (Klimstra et al., 2009; Gunther Moor et al., 2010). Our results are also in agreement with the finding in humans that social seeking is low in early adolescence (Dubey et al., 2017) and additionally indicate that such a result may actually reflect that *the surveyed group had met basic belongingness needs*, making such findings applicable to a part rather than to all early-adolescents (which to some extent explains the discrepancy in the results of studies examining the developmental course of sociability). The results of the present study are also useful in the context of neurodevelopmental imbalance and evolutionary models of adolescent risky decision-making, with the former arguing that peer presence causes increased risk-taking, particularly during early adolescence (Crone and Dahl, 2012), and the latter suggesting that effects would be the most pronounced in males for both early- and mid-adolescents (Defoe et al., 2020). Motivational arousal due to peer presence could be expected in those who grow up in a social environment that does not support their social needs, particularly in early adolescence, so *previous social experience in addition to age* (Crone and Dahl, 2012) *and gender* (Defoe et al., 2020) *should be taken into consideration when analyzing the influence of “the mere peer presence” in adolescence.* This presumption is in agreement with the findings that the promotion of social wanting and the activation of the motivational/dopaminergic reward system in the midbrain happen in the presence of social cues in isolated both mice (Matthews et al., 2016) and humans (Tomova et al., 2020) and that a hyper-responsive motivational-reward system can undermine self-regulation in the presence of peers (Albert et al., 2013).

### Limitation

Although the current study has notable strength, revealing important details due to the particular methodological approach (Templer et al., 2018), the assessment of tightly coupled behavioral manifestations of social motivation and cognition (Chevallier et al., 2012), the estimation of maturational response to the social olfactory cues as an important component of approach behavior (Haskal et al., 2020; Contestabile et al., 2021), and the analysis of the PVI-labeled neurons in the dorsal hippocampus that is involved in socio-spatial navigation (Tavares et al., 2015), it could be improved by the addition of findings in female rats. Consequently, the absence of such findings could be considered a limitation of the study.

## Conclusion

This study accentuates the maturational complexity of motivated social behavior as well as the power of reduced direct social contact with peers in adolescence to violate this course at different functional levels, thus bringing the autograph specific to the period when the experience occurred. Moreover, it helps to understand the sculpting influence of the appetitive and consummatory aspects of sociability on social cognition, identify cross-species similarities in the maturation of motivated social behavior, and extend the knowledge of the functioning of the rodent model as such. Overall, the obtained data emphasizes the biological importance of meeting social needs in adolescence in regulating processes that underlie the maturation of motivated social behavior and cognition in social mammals.

## Data Availability Statement

The original contributions presented in the study are included in the article/Supplementary Material, further inquiries can be directed to the corresponding author.

## Ethics Statement

The animal study was reviewed and approved by the Ethical Committee of the Institute for Biological Research “Siniša Stanković” - National Institute of Republic of Serbia (approval number: 01-01/19) and the National Ethics Research Committee (Veterinary Directorate of the Republic of Serbia; approval number: 323-07-05339/2020-05).

## Author Contributions

MP and VP contributed to the study design, analysis and interpretation of data, and manuscript writing. MP, ŽP, and NP contributed to the acquisition of data (experimental work). NP contributed to the antibodies availability. ŽP and MP contributed to the data visualization. All authors have read and approved the final version of this article.

## Conflict of Interest

The authors declare that the research was conducted in the absence of any commercial or financial relationships that could be construed as a potential conflict of interest.

## Publisher’s Note

All claims expressed in this article are solely those of the authors and do not necessarily represent those of their affiliated organizations, or those of the publisher, the editors and the reviewers. Any product that may be evaluated in this article, or claim that may be made by its manufacturer, is not guaranteed or endorsed by the publisher.

## Supplementary Material

Supplementary Table S1 The summary of statistical analysis used in the study.
