# Supplementary material for: The Influence of Social Isolation on Social Orientation, Sociability, Social Novelty Preference, and Hippocampal Parvalbumin-Expressing Interneurons in Peripubertal Rats – Understanding the Importance of Meeting Social Needs in Adolescence
Source: Front Behav Neurosci. 2022 May 3;16:872628. doi: 10.3389/fnbeh.2022.872628 (PMC9113078; doi:10.3389/fnbeh.2022.872628)
Supplement: Supplementary Table S1 — The summary of statistical analysis used in the study. [file Table_1.pdf]

Supplementary Table 1 (S1).

Figure 3A

	Factor: Maturation under given housing conditions early- vs. mid-adolescence, Wilcoxon test					Factor: Housing conditions GH vs SH, Mann Whitney U-test			
		N	T	Z	p		U	Z	p
Number of entries in social section (with novel conspecific 1; **N1**)	GH	7	9.5	0.210	0.834	early	15	1.214	0.225
	SH	7	7.5	0.629	0.529	mid	18.5	0.767	0.443
Number of entries in non-social section (with object; **O**)	GH	7	8.5	0.419	0.675	early	14.5	-1.278	0.201
	SH	7	8	1.014	0.310	mid	20	0.575	0.565
Total number of entries in sections (**N1** + **O**)	GH	7	10	0.105	0.917	early	21	0.447	0.655
	SH	7	10.5	0.592	0.554	mid	20.5	0.511	0.609

Figure 3B

	Two-way repeated measures ANOVA	Tukey (HSD)	
Preference based on number of entries in sections (**DInes**)	Factor: Housing F (1,12) = 5.852, p = 0.032	GH early vs GH mid	p = 0.784
	Factor: Maturation F (1,12) = 0.001, p = 0.982	SH early vs SH mid	p = 0.766
	Factor: Maturation x Housing F (1,12) = 1.836, p = 0.200	GH early vs SH early	p = 0.112
		GH mid vs SH mid	p = 0.788

Figure 3C

		Early-adolescence			Mid-adolescence		
Time spent in social section (**N1**) vs. time spent in center (**C**) (t test for dependent samples)		t	df	p	t	df	p
	GH	6.232	6	<0.001	5.927	6	<0.001
	SH	7.359	6	<0.001	6.922	6	<0.001
Time spent in social section (**N1**) vs. time spent in section with object (**O**) (t test for dependent samples)		t	df	p	t	df	p
	GH	0.194	6	0.852	1.835	6	0.116
	SH	2.455	6	0.049	2.864	6	0.029

	Two-way repeated measures ANOVA	Post hoc test
Time spent in social section (**N1**)	Factor: Housing F (1,12) = 1.705, p = 0.216<br>Factor: Maturation F (1,12) = 1.888, p = 0.195<br>Factor: Maturation x Housing F (1,12) = 0.432, p = 0.524	not applicable
Time spent in center (**C**)	Factor: Housing F (1,12) = 0.851, p = 0.375<br>Factor: Maturation F (1,12) = 0.559, p = 0.469<br>Factor: Maturation x Housing F (1,12) = 0.066, p = 0.801	not applicable
Time spent in section with object (**O**)	Factor: Housing F (1,12) = 2.385, p = 0.148<br>Factor: Maturation F (1,12) = 1.538, p = 0.239<br>Factor: Maturation x Housing F (1,12) = 0.608, p = 0.451	not applicable

Figure 4A

	Two-way repeated measures ANOVA	Tukey (HSD)	
Number of approaches to novel conspecific 1 (**N1**)	Factor: Housing F (1,12) = 1.498, p = 0.244<br>Factor: Maturation F (1,12) = 1.831, p = 0.201<br>Factor: Maturation x Housing F (1,12) = 0.011, p = 0.919	Post hoc test not applicable	
Number of approaches to object (**O**)	Factor: Housing F (1,12) = 4.280, p = 0.061<br>Factor: Maturation F (1,12) = 7.107, p = 0.021<br>Factor: Maturation x Housing F (1,12) = 5.832, p = 0.033	GH early vs GH mid	p = 0.017
		SH early vs SH mid	p = 0.998
		GH early vs SH early	p = 0.068
		GH mid vs SH mid	p = 0.999
Total number of approaches (**N1** + **O**)	Factor: Housing F (1,12) = 0.570, p = 0.465<br>Factor: Maturation F (1,12) =10.600, p = 0.007<br>Factor: Maturation x Housing F (1,12) = 3.705, p = 0.078	GH early vs GH mid	p = 0.015
		SH early vs SH mid	p = 0.784
		GH early vs SH early	p = 0.301
		GH mid vs SH mid	p = 0.864

Figure 4B

	Factor: Maturation under given housing conditions early- vs. mid-adolescence, Wilcoxon test					Factor: Housing conditions GH vs SH, Mann Whitney *U*-test			
		N	T	Z	p		U	Z	p
Preference based on number of approaches (**DIna**)	GH	7	8	1.014	0.310	early	7	2.236	0.025
	SH	7	14	0	1	mid	15	1.214	0.225

Figure 4C

	Two-way repeated measures ANOVA					Tukey (HSD)			
Time spent in novel conspecific 1 (**N1**) exploration	**Factor: Maturation** early- vs. mid-adolescence, Wilcoxon test					**Factor: Housing conditions** GH vs SH, Mann Whitney *U*-test			
		N	T	Z	p		U	Z	p
	GH	7	2	2.028	0.043	early	17	0.958	0.338
	SH	7	5	1.153	0.249	mid	9.5	1.917	0.055

	Two-way repeated measures ANOVA	Tukey (HSD)	
Time spent in object (**O**) exploration	Factor: Housing F (1,12) = 3.023, p = 0.108<br>Factor: Maturation F (1,12) = 18.494, p = 0.001<br>Factor: Maturation x Housing F(1,12) = 16.008, p = 0.002	GH early vs GH mid	p = 0.001
		SH early vs SH mid	p = 0.997
		GH early vs SH early	p = 0.107
		GH mid vs SH mid	p = 1.000
Total exploratory time (**N1** + **O**)	Factor: Housing F (1,12) = 0.398, p = 0.540<br>Factor: Maturation F (1,12) = 16.014, p = 0.002<br>Factor: Maturation x Housing F (1,12) = 1.238, p = 0.288	GH early vs GH mid	p = 0.016
		SH early vs SH mid	p = 0.226
		GH early vs SH early	p = 0.999
		GH mid vs SH mid	p = 0.737

Figure 4D

	Two-way repeated measures ANOVA	Tukey (HSD)	
Preference based on exploratory time (**DIet**)	Factor: Housing F (1,12) = 11.759, p = 0.005<br>Factor: Maturation F (1,12) = 0.437, p = 0.521<br>Factor: Maturation x Housing F (1,12) = 1.959, p = 0.187	GH early vs GH mid	p = 0.491
		SH early vs SH mid	p = 0.952
		GH early vs SH early	p = 0.023
		GH mid vs SH mid	p = 0.503

Figure 5A

	Two-way repeated measures ANOVA	Tukey (HSD)	
Number of entries in section with familiar conspecific (**N1**)	Factor: Housing F (1,11) = 14.442, p = 0.003	GH early vs GH mid	p = 0.918
	Factor: Maturation F (1,11) = 0.804, p = 0.389	SH early vs SH mid	p = 0.243
	Factor: Maturation x Housing F (1,11) = 3.360, p = 0.094	GH early vs SH early	p = 0.001
		GH mid vs SH mid	p = 0.998
Number of entries in section with novel conspecific (**N2**)	Factor: Housing F (1,11) = 0.179, p = 0.680<br>Factor: Maturation F (1,11) = 0.460, p = 0.512<br>Factor: Maturation x Housing F (1,11) = 0.827, p = 0.383	Post hoc test not applicable	
Total number of entries in both sections (**N1 + N2**)	Factor: Housing F (1,11) = 5.788, p = 0.035	GH early vs GH mid	p = 0.970
	Factor: Maturation F (1,11) = 0.696, p = 0.422	SH early vs SH mid	p = 0.366
	Factor: Maturation x Housing F (1,11) = 2.199, p = 0.166	GH early vs SH early	p = 0.014
		GH mid vs SH mid	p = 0.953

Figure 5B

	Two-way repeated measures ANOVA	Post hoc test
Preference based on number of entries in sections (**DInes**)	Factor: Housing F (1,11) = 1.105, p = 0.316<br>Factor: Maturation F (1,11) = 0.000, p = 0.996<br>Factor: Maturation x Housing F (1,11) = 1.011, p = 0.336	not applicable

Figure 5C

		early-adolescence				mid-adolescence			
Time spent in **N2** section vs. time spent in center (**C**), Wilcoxon test		N	T	Z	p	N	T	Z	p
	GH	6	2	1.782	0.075	6	0	2.201	0.028
	SH	7	2	0.028	0.043	7	1	2.197	0.028
Time spent in section with social novelty (**N2**) vs. time spent in section with social familiarity (**N1**), Wilcoxon test		N	T	Z	p	N	T	Z	p
	GH	6	2	1.782	0.075	6	3	1.572	0.116
	SH	7	2	0.028	0.043	7	10.5	0.592	0.554

Time spent in section with social familiarity (**N1**)			early-adolescence			mid-adolescence		
	**Factor: Housing condition** GH vs. SH, Mann Whitney U-test		U	Z	p	U	Z	p
			6	-2.143	0.032	19	-0.286	0.775

Time spent in section with social familiarity (**N1**)			early vs. mid-adolescence			
	**Factor: Maturation**		N	T	Z	p
	Wilcoxon test	GH	6	8	0.524	0.600
		SH	7	9	0.485	0.398

Time spent in center (**C**)		early-adolescence			mid-adolescence		
**Factor: Housing condition** GH vs. SH, Mann Whitney U-test		U	Z	p	U	Z	p
		9.5	-1.643	0.100	6.5	2.071	0.038

Time spent in center (**C**)		early- vs. mid-adolescence			
**Factor: Maturation**		N	T	Z	p
Wilcoxon test	GH	6	6	0.943	0.345
Wilcoxon test	SH	7	6	1.352	0.176

Time spent in section with social novelty (**N2**)		early-adolescence			mid-adolescence		
**Factor: Housing condition** GH vs. SH, Mann Whitney U-test		U	Z	p	U	Z	p
		11	1.429	0.153	20	0.143	0.886

Time spent in section with social novelty (**N2**)		early- vs. mid-adolescence			
**Factor: Maturation**		N	T	Z	p
Wilcoxon test	GH	6	10	0.105	0.197
Wilcoxon test	SH	7	7	0.183	0.237

Figure 6A

	Two-way repeated measures ANOVA	Post hoc test
Number of approaches to familiar conspecific (**N1**)	Factor: Housing F (1,11) = 4.476, p = 0.058<br>Factor: Maturation F (1,11) = 4.227, p = 0.064<br>Factor: Maturation x Housing F (1,11) = 4.227, p = 0.064	not applicable
Number of approaches to novel conspecific (**N2**)	Factor: Housing F (1,11) = 1.959, p = 0.189<br>Factor: Maturation F (1,11) = 0.075, p = 0.789<br>Factor: Maturation x Housing F (1,11) = 0.165, p = 0.692	not applicable
Total number of approaches (**N1** + **N2**)	Factor: Housing F (1,11) = 0.168, p = 0.690<br>Factor: Maturation F (1,11) = 3.866, p = 0.075<br>Factor: Maturation x Housing F (1,11) = 4.320, p = 0.062	not applicable

Figure 6B

	Two-way repeated measures ANOVA	Post hoc test
Preference based on number of approaches (**DIna**)	Factor: Housing F (1,11) = 3.254, p = 0.099<br>Factor: Maturation F (1,11) = 0.967, p = 0.347<br>Factor: Maturation x Housing F (1,11) = 0.212, p = 0.654	not applicable

Figure 6C

	Two-way repeated measures ANOVA	Tukey (HSD)	
Time spent in **N1**exploration	Factor: Housing F (1,11) = 9.590, p = 0.010<br>Factor: Maturation F (1,11) = 1.526, p = 0.242<br>Factor: Maturation x Housing F (1,11) = 2.250, p = 0.162	GH early vs GH mid	p = 0.998
		SH early vs SH mid	p = 0.241
		GH early vs SH early	p = 0.017
		GH mid vs SH mid	p = 0.946
Time spent in **N2** exploration	Factor: Housing F (1,11) = 0.018, p = 0.897<br>Factor: Maturation F (1,11) = 0.006, p = 0.939<br>Factor: Maturation x Housing F (1,11) = 0.026, p = 0.875	Post hoc test not applicable	

Total exploratory time (**N1** + **N2**)	Factor: Housing F (1,11) = 2.056, p = 0.179<br>Factor: Maturation F (1,11) = 0.673, p = 0.429<br>Factor: Maturation x Housing F (1,11) = 1.433, p = 0.256	Post hoc test not applicable

Figure 6D

	Two-way repeated measures ANOVA	Post hoc test
Preference based on exploratory time (**DIet**)	Factor: Housing F (1,11) = 4.695, p = 0.053<br>Factor: Maturation F (1,11) = 0.665, p = 0.432<br>Factor: Maturation x Housing F (1,11) = 0.251, p = 0.626	not applicable

Figure 7A

	Two-way repeated measures ANOVA	Tukey (HSD)	
Number of approaches to a box with social odor 1 (**S1**)	Factor: Housing F (1,14) = 0.041, p = 0.843<br>Factor: Maturation F (1,14) = 6.723, p = 0.021<br>Factor: Maturation x Housing F (1,14) = 3.478, p = 0.083	GH early vs GH mid	p = 0.954
		SH early vs SH mid	p = 0.032
		GH early vs SH early	p = 0.763
		GH mid vs SH mid	p = 0.898

	Factor: Maturation under given housing conditions early- vs. mid-adolescence, Wilcoxon test					Factor: Housing conditions GH vs SH, Mann Whitney U-test			
		N	T	Z	p		U	Z	p
Number of approaches to a box with no smell (**NS**)	GH	8	10	0.676	0.499	early	22	-1.05	0.294
	SH	8	9.5	1.190	0.234	mid	20	-1.26	0.208

	Two-way repeated measures ANOVA	Tukey (HSD)	
Total number of approaches (**S1 + NS**)	Factor: Housing F (1,14) = 0.623, p = 0.443<br>Factor: Maturation F (1,14) = 4.718, p = 0.048<br>Factor: Maturation x Housing F (1,14) = 0.590, p = 0.455	GH early vs GH mid	p = 0.756
		SH early vs SH mid	p = 0.208
		GH early vs SH early	p = 1.000
		GH mid vs SH mid	p = 0.719

Figure 7B

	Factor: Maturation under given housing conditions Early- vs. mid-adolescence, Wilcoxon test					Factor: Housing conditions GH vs SH, Mann Whitney U-test			
		N	T	Z	p		U	Z	p
Preference based on number of approaches (**DIna**)	GH	8	12	0.840	0.401	early	13	1.995	0.046
	SH	8	10	0.676	0.499	mid	30	0.210	0.834

Figure 7C

	Two-way repeated measures ANOVA	Tukey (HSD)	
Time spent in exploration of a box with social odor 1 (**S1**)	Factor: Housing F (1,14) = 3.864, p = 0.070<br>Factor: Maturation F (1,14) = 10.860, p = 0.005<br>Factor: Maturation x Housing F (1,14) = 5.913, p = 0.029	GH early vs GH mid	p = 0.927
		SH early vs SH mid	p = 0.006
		GH early vs SH early	p = 0.049
		GH mid vs SH mid	p = 0.999
Time spent in exploration of a box with no smell (**NS**)	Factor: Housing F (1,14) = 1.391, p = 0.258<br>Factor: Maturation F (1,14) = 10.971, p = 0.005<br>Factor: Maturation x Housing F (1,14) = 0.040, p = 0.844	GH early vs GH mid	p = 0.106
		SH early vs SH mid	p = 0.171
		GH early vs SH early	p = 0.769
		GH mid vs SH mid	p = 0.896

Total exploratory time (**S1** + **NS**)	Factor: Housing F (1,14) = 0.478, p = 0.402	GH early vs GH mid	p = 0.490
	Factor: Maturation F (1,14) = 15.381, p = 0.002	SH early vs SH mid	p = 0.005
	Factor: Maturation x Housing F (1,14) = 3.488, p = 0.083	GH early vs SH early	p = 0.301
		GH mid vs SH mid	p = 0.928

Figure 7D

	Factor: Maturation under given housing conditions early- vs. mid-adolescence, Wilcoxon test					Factor: Housing conditions GH vs SH, Mann Whitney U-test			
		N	T	Z	p		U	Z	p
Preference based on exploratory time (**DIet**)	GH	8	14	0.560	0.575	early	9	2,415	0.016
	SH	8	3	2.100	0.036	mid	27	0,525	0.600

Figure 8A

	Two-way repeated measures ANOVA	Post hoc test
Number of approaches to a box with familiar social odor (**S1**)	Factor: Housing F (1,14) = 2.168, p = 0.163<br>Factor: Maturation F (1,14) = 0.107, p = 0.748<br>Factor: Maturation x Housing F (1,14) = 4.834, p = 0.055	not applicable
Number of approaches to a box with novel social odor (**S2**)	Factor: Housing F (1,14) = 0.305, p = 0.590<br>Factor: Maturation F (1,14) = 0.888, p = 0.362<br>Factor: Maturation x Housing F (1,14) = 0.284, p = 0.603	not applicable
Total number of approaches (**S1** + **S2**)	Factor: Housing F (1,14) = 2.028, p = 0.176<br>Factor: Maturation F (1,14) = 0.671, p = 0.426<br>Factor: Maturation x Housing F (1,14) = 0.862, p = 0.369	not applicable

Figure 8B

	Two-way repeated measures ANOVA	Post hoc test
Preference based on number of approaches (**DIna**)	Factor: Housing F (1,14) = 0.340, p = 0.569<br>Factor: Maturation F (1,14) = 0.134, p = 0.720<br>Factor: Maturation x Housing F (1,14) = 3.932, p = 0.067	not applicable

Figure 8C

	Two-way repeated measures ANOVA	Post hoc test
Time spent in exploration of a box with familiar social odor (**S1**)	Factor: Housing F (1,14) = 1.036, p = 0.326<br>Factor: Maturation F (1,14) = 0.048, p = 0.830<br>Factor: Maturation x Housing F (1,14) = 2.194, p = 0.161	not applicable

	Factor: Maturation under given housing conditions early- vs. mid-adolescence, Wilcoxon test					Factor: Housing conditions GH vs SH, Mann Whitney U-test			
		N	T	Z	p		U	Z	p
Time spent in exploration of a box with novel social odor (**S2**)	GH	8	5.5	1.750	0.080	early	20.5	1.208	0.227
	SH	8	6	1.680	0.093	mid	22.5	0.998	0.318

Total exploratory time (**S1** + **S2**)	GH	8	11.5	0.910	0.363	early	17.5	1.523	0.128
	SH	8	6	1.680	0.093	mid	27	0.525	0.600

Figure 8D

Preference based on exploratory time (**DIet**)	Factor: Housing F (1,14) = 0.400, p = 0.537	GH early vs GH mid	p = 0.186
	Factor: Maturation F (1,14) = 5.497, p = 0.034	SH early vs SH mid	p = 0.657
	Factor: Maturation x Housing F (1,14) = 0.483, p = 0.499	GH early vs SH early	p = 0.780
		GH mid vs SH mid	p = 1.000

Figure 9C

	Factor: Maturation under given housing conditions early- vs. mid-adolescence, Mann Whitney U-test				Factor: Housing conditions GH vs SH, Mann Whitney U-test			
		T	Z	p		U	Z	p
Neurons with PV+ immunoreactivity in CA1 (number per section/side/brain)	GH	8	0	1	early	7	0.289	0.773
	SH	5	-0.866	0.386	mid	8	0	1

Figure 9D

	Factor: Maturation under given housing conditions early- vs. mid-adolescence, Mann Whitney U-test				Factor: Housing conditions GH vs SH, Mann Whitney U-test			
		T	Z	p		U	Z	p
Neurons with PV+ immunoreactivity in CA2 (number per section/side/brain)	GH	5	0.866	0.386	early	4	-1.155	0.248
	SH	7	-0.289	0.773	mid	6.5	-0.433	0.665

Figure 9E

	Factor: Maturation under given housing conditions early- vs. mid-adolescence, Mann Whitney U-test				Factor: Housing conditions GH vs SH, Mann Whitney U-test			
		T	Z	p		U	Z	p
Neurons with PV+ immunoreactivity in CA3 (number per section/side/brain)	GH	1	-2.021	0.043	early	7.5	-0.144	0.885
	SH	0	-2.309	0.021	mid	5	0.866	0.386

Figure 10B

	Factor: Maturation under given housing conditions early- vs. mid-adolescence, Mann Whitney U-test				Factor: Housing conditions GH vs SH, Mann Whitney U-test			
		T	Z	p		U	Z	p
Neurons with PV+ immunoreactivity in DG (number per section/side/brain)	GH	4.5	1.010	0.312	early	1	2.021	0.043
	SH	4	-1.155	0.248	mid	4	1.155	0.248
